# Plant compartment and biogeography affect microbiome composition in cultivated and native *Agave* species

**DOI:** 10.1111/nph.13697

**Published:** 2015-10-15

**Authors:** Devin Coleman‐Derr, Damaris Desgarennes, Citlali Fonseca‐Garcia, Stephen Gross, Scott Clingenpeel, Tanja Woyke, Gretchen North, Axel Visel, Laila P. Partida‐Martinez, Susannah G. Tringe

**Affiliations:** ^1^ Department of EnergyJoint Genome InstituteWalnut CreekCA94598USA; ^2^ Genomics DivisionLawrence Berkeley National LaboratoryBerkeleyCA94720USA; ^3^ Plant Gene Expression CenterUSDA‐ARSAlbanyCA94710USA; ^4^ Departamento de Ingeniería GenéticaCentro de Investigación y de Estudios AvanzadosIrapuato36821Mexico; ^5^ Department of BiologyOccidental CollegeLos AngelesCA90041USA; ^6^ School of Natural SciencesUniversity of CaliforniaMercedCA95343USA

**Keywords:** *Agave*, biogeography, cultivation, desert, iTags, microbial diversity, plant microbiome, plant–microbe interactions

## Abstract

- Desert plants are hypothesized to survive the environmental stress inherent to these regions in part thanks to symbioses with microorganisms, and yet these microbial species, the communities they form, and the forces that influence them are poorly understood.
- Here we report the first comprehensive investigation of the microbial communities associated with species of *Agave*, which are native to semiarid and arid regions of Central and North America and are emerging as biofuel feedstocks. We examined prokaryotic and fungal communities in the rhizosphere, phyllosphere, leaf and root endosphere, as well as proximal and distal soil samples from cultivated and native agaves, through Illumina amplicon sequencing.
- Phylogenetic profiling revealed that the composition of prokaryotic communities was primarily determined by the plant compartment, whereas the composition of fungal communities was mainly influenced by the biogeography of the host species. Cultivated *A. tequilana* exhibited lower levels of prokaryotic diversity compared with native agaves, although no differences in microbial diversity were found in the endosphere.
- Agaves shared core prokaryotic and fungal taxa known to promote plant growth and confer tolerance to abiotic stress, which suggests common principles underpinning *Agave*–microbe interactions.

## Abstract 2

See also the Commentary by Hacquard

## Introduction

The genus *Agave*, native to the deserts and dry lands of central Mexico and the southwestern USA, includes a number of promising candidate biofuel crops for arid and semiarid climates (Somerville *et al*., 2010; Davis *et al*., 2011). There are > 200 species of *Agave*, and several of these have been cultivated for centuries for the production of alcohols and fiber (Garcia‐Moya *et al*., 2011). Recent research has highlighted the suitability of agaves as biofuel feedstocks as a consequence of their comparatively low lignin content, ease of decomposition, and high yields with minimal resource inputs (Davis *et al*., 2014; Li *et al*., 2014). Additionally, agaves are capable of growing on otherwise nonarable rocky soils in regions characterized by prolonged drought and extreme temperatures owing in part to physiological and biochemical adaptations that prevent excess water loss (Davis *et al*., 2011; Shakeel *et al*., 2012; Campos *et al*., 2014). This ability to grow on subprime land reduces the competition for valuable and limited arable agricultural land between *Agave* and other food crops (Borland *et al*., 2009; Holtum *et al*., 2011).

However, the economic viability of *Agave*‐based biofuels will hinge on the maximization of plant yield (Núñez *et al*., 2011). Conventional methods of crop improvement with agaves are possible, but are made more difficult by the long period plants take to reach maturity; transgenic approaches could accelerate this process but have been impeded by the limited tools for genetic transformation of *Agave* (Flores‐Benítez *et al*., 2007; Escamilla‐Treviño, 2012).

It is now well established that symbiotic bacteria and fungi have profound impacts on plant health and adaptation to stress (Lugtenberg & Kamilova, 2009; Partida‐Martínez & Heil, 2011; Gaiero *et al*., 2013; Mendes *et al*., 2013; Philippot *et al*., 2013; Panke‐Buisse *et al*., 2015) and that manipulation of the plant microbiome has the potential to dramatically improve the yield of agronomically important crops (Turner *et al*., 2013). Indeed, it has been proposed that plants should be considered a collective ‘holobiont’ rather than standalone entities (Zilber‐Rosenberg & Rosenberg, 2008; Vandenkoornhuyse *et al*., 2015). Recent research has demonstrated that plant‐growth‐promoting microbes (PGPM) harvested from plants grown in semiarid environments are capable of enhancing plant growth and stress tolerance in a variety of crop species (Marasco *et al*., 2012; Mengual *et al*., 2014; Rolli *et al*., 2015). In *Agave*, recent research highlighted the likely beneficial role of native associated diazotrophic bacteria, which may play a role not only in nutrition, but also in tolerance to drought (Desgarennes *et al*., 2014). Also, previous reports in which either mycorrhizal or endophytic fungi were found to promote plant growth in *Agave* species (Cui & Nobel, 1992; Obledo *et al*., 2003) suggest that the *Agave* microbiome plays an important role in the fitness and productivity of this genus. Thus, an alternative to improve *Agave*'s yield may be the rational use and manipulation of the microbiome.

Unlike many food crops, current commercially important *Agave* species and cultivars have undergone limited domestication efforts, despite having been grown under continuous cultivation for centuries in plantations that exist in proximity to related native populations (Nobel, 2003). Therefore, the genus also offers an opportunity to evaluate whether cultivation is associated with changes in the composition of microbial communities associated with a plant host, as has been suggested for other species by recent studies (Edwards *et al*., 2015). Importantly, studies in other plants and soil systems have provided divergent results, describing examples of both decreased (Fierer *et al*., 2013) and increased microbial diversity (Rodrigues *et al*., 2013) upon cultivation.

Previous studies investigating the microbial communities associated with diverse plant hosts have demonstrated that sample type (plant compartment), host species, geographic location, and season are all capable of influencing community composition, although the extent to which each plays a role has varied from study to study (DeAngelis *et al*., 2008; Lundberg *et al*., 2012; Lançoni *et al*., 2013; Peiffer *et al*., 2013; Mendes *et al*., 2014; Edwards *et al*., 2015; Zarraonaindia *et al*., 2015). Most previous studies have focused on prokaryotic communities, providing limited insights into the forces shaping plant‐associated fungal communities. While recent comparative studies (Hilton *et al*., 2013; Shakya *et al*., 2013) indicate that distinct factors can drive fungal and prokaryotic rhizosphere communities, it is unclear to what extent these differences extend to other plant compartments, plant hosts and soil systems.

**Figure 1:**
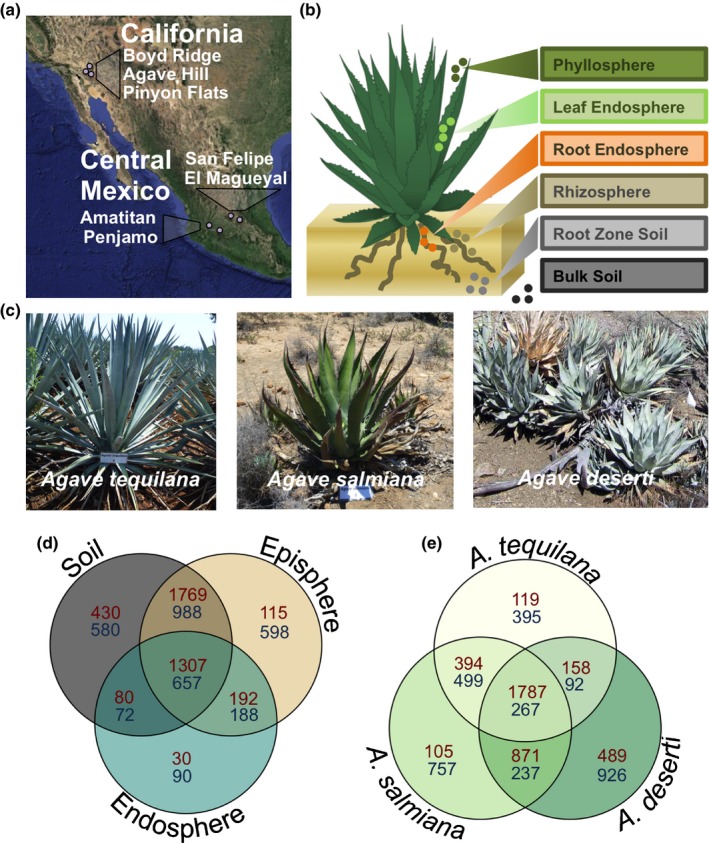
Experimental design of this study. (a) Study sites and biogeography of selected *Agave* species. (b) The six samples analyzed from each plant. (c) Pictures of *A. tequilana*,* A. salmiana* and *A. deserti*. (d, e) Venn diagrams of shared prokaryotic (red) and fungal (blue) operational taxonomic units across groups of sample types and across *Agave* species.

The primary goal of this research was to investigate the prokaryotic and fungal communities associated with the bulk and proximal soil, the rhizosphere, the phyllosphere, and the root and leaf endospheres, for three *Agave* species: the cultivated *Agave tequilana* and the native species, *Agave salmiana* and *Agave deserti* (Fig. 1a–c). These species were selected based on their natural geographic distribution (Table 1; Fig. 1a) (CONABIO, 2006; Gentry *et al*., 2013), their differentiated management status (cultivated versus native), and their relevance to both existing and prospective industries. Comparisons across these three *Agave* species would allow us to infer the impact of biogeography and cultivation status on microbial composition and diversity in the genus *Agave*. Additionally, we also evaluate the major biotic (i.e. plant species and plant compartment) and abiotic (i.e. season and site) factors shaping both prokaryotic and fungal communities, and identify prominent microbial players in order to develop rational microbiome‐based strategies for improving the yield and productivity of *Agave*.

**Table 1:** Geographic location, annual mean temperature, precipitation and soil characteristics of study sites

		Sites						
		Mexico				USA		
		Jalisco	Guanajuato			California		
		*Agave tequilana*		*A. salmiana*		*A. deserti*		
		Amatitan (Am)	Penjamo (Pe)	El Magueyal (Ma)	San Felipe (SF)	Boyd Ridge (BR)	Agave Hill (AH)	Pinyon Flats (PF)
Latitude		21.053	20.686	21.195	21.766	33.714	33.665	33.601
Longitude		−103.902	−101.875	−100.439	−100.163	−116.524	−116.425	−116.595
Altitude (m above sea level)		1260	1714	2175	2089	452	814	1225
Annual mean temperature (°C)^a^		26.4	19	17.9	17	23.7	20.6	18.0
Annual precipitation (mm)^a^		558	790	485	204.5	136.0	183.0	238.3
Precipitation during dry season (mm)^a^		4.2	147	130	68	51.7	69.4	95.5
Precipitation during rainy season (mm)^a^		553.8	643	355	136.5	83.9	113.6	142.8
Soil edaphic factors	Texture	Clay loam	Clay	Sandy loam	Sandy loam	Sandy loam	Sandy loam	Sandy loam
	pH^b^	6.8	6.68	5.79	6.25	8.02	7.48	7.95
	Organic matter (%)	1.1	0.96	3.97	0.71	nd	nd	nd
	Nitrogen (μg g^−1^)	19.7	9.14	8.76	12.65	nd	nd	nd
	Phosphorus (μg g^−1^)	3.64	27.1	18.11	4.53	42.17	87	74
	Potassium (μg g^−1^)	193	482	64.35	251.7	155.5	131	91.33

a For sites in Mexico, data were provided by Comisión Nacional del Agua (CONAGUA). Data from sites in California were obtained from DRI Weather Station Data Collection (http://www.wrcc.dri.edu/weather/ucde.html). nd, not determined.

b Statistically significant difference between species after Kruskal–Wallis test (*H*_*2,*15_ = 10.763; *P *= 0.0046).

## Materials and Methods

### Experimental design

Our study investigated three *Agave* species: *Agave tequilana* F.A.C. Weber, *Agave salmiana* Otto ex Salm subsp. *crassispina* (Trel.) Gentry, and *Agave deserti* Engelm. These species are distributed in central Mexico and in southern California, but they are not sympatric (Fig 1a). *Agave tequilana* belongs to the group *Rigidae* (Gentry, 1982) and exists only in cultivated populations, mainly in Jalisco (Fig. 1a). *Agave salmiana* belongs to the group *Salmianae*, whose natural populations extend from Sonora to Guerrero in western Mexico (Gentry, 1982; CONABIO, 2006). *Agave deserti* belongs to the group *Deserticolae* and it is native to California and can also be found in parts of Arizona and Baja California (Gentry, 1982). In 2012, samples were collected at two different times: in late May (the end of the 8 months of low or null precipitation in Mexico) and in early October (at the end of the 4 months of the most precipitation in Mexico). *Agave tequilana* samples were taken from two fields managed by tequila companies in Guanajuato and Jalisco, Mexico: Penjamo (Pe) and Amatitan (Am) (Fig. 1a), while *Agave salmiana* samples were collected from the natural populations of El Magueyal (Ma) and San Felipe (SF) in Guanajuato, Mexico. *Agave deserti* samples were collected in September and April (in a year with little rainfall) from the natural populations within the Philip L. Boyd Deep Canyon Desert Reserve in California called Agave Hill (AH), Boyd Ridge (BR), and Pinyon Flats (PF). Soil characteristics as well as temperature and precipitation data for each site are presented in Table 1. For each species, and at each location and time, samples from three healthy plants were collected. Four samples per plant were collected to analyze six different communities (Fig. 1b): two leaves for leaf endosphere and phyllosphere; root tissue for root endosphere and rhizosphere; root zone soil, which was soil loosely attached to or adjacent to the roots, and bulk soil gathered 1 m away from the selected plant specimen. All samples were stored at 4°C until processing. We analyzed in total 252 samples; 72 from *A. tequilana*, 72 from *A. salmiana* and 108 from *A. deserti*.

### Sample preparation, DNA extraction, PCR, and sequencing

Samples were prepared and total DNA was extracted as previously described (Desgarennes *et al*., 2014). For amplification of the ITS2 and 16S regions, we used the ITS9F/ITS4R and 515F/816R primer pairs, respectively, and followed the PCR protocol described in the Supporting Information. For 16S amplification, peptide nucleic acid (PNA) clamps were used as in Lundberg *et al*. (2013), which substantially reduced overall chloroplast and mitochondrial contamination and increased the proportion of prokaryotic reads for the root endosphere, leaf endosphere, and phyllosphere samples. Paired‐end 2 × 250 bp sequencing was performed on an Illumina MiSeq instrument (Illumina Inc., San Diego, CA, USA) operating with v2 chemistry. All quality sequences related to this project are available in the NCBI Sequence Read Archive (SRA) under project IDs SRA211411, SRA211420, SRA211416, SRA211422 and SRA211408.

### Data processing and statistical analyses

The raw Fastq reads were processed using a custom pipeline developed at the Joint Genome Institute (Tremblay *et al*., 2015) (Supporting Information Methods S1). Raw reads were contaminant‐filtered, quality trimmed, merged and clustered to produce 25 871 and 40 759 fungal and prokaryotic operational taxonomic units (OTUs), respectively, at 95% and 97% identity using the UPARSE pipeline (Edgar, 2013). Taxonomies were assigned to each OTU using the RDP Naïve Bayesian Classifier (Wang *et al*., 2007) with custom reference databases. OTUs whose RDP classifications did not match their expected taxonomic kingdoms (Fungi and Bacteria/Archaea, respectively) were removed. Average read counts varied by sample type for both data sets, and in particular the aerial endophytic samples had substantially fewer reads than the other sample types (Fig. S1). To reduce low‐abundance and spurious OTUs, technical reproducibility thresholds determined empirically from technical replicates as in Lundberg *et al*. (2013) (Fig. S2) were set and OTUs kept only if they had at least two reads in at least five samples (ITS2 data) or at least seven reads in at least five samples (16S data).

For diversity analyses, all samples were randomly subsampled (rarefied) to 1000 reads per sample to account for differences in the number of reads across samples. We calculated the Shannon diversity (H′) index using the package ‘BiodiversityR’ in R. All other statistical analyses were performed in R using a variety of packages and custom scripts using the measurable OTUs, as greater statistical support was achieved using this data set instead of the measurable rarefied data set. In brief, Venn diagrams were plotted with the function ‘venn.diagram’ using the package ‘VennDiagram’. Distances were calculated using the ‘vegdist’ function of the package ‘vegan’ for Bray–Curtis. Nonmetric multidimensional scaling (NMDS) was performed using ‘Mass’ and ‘vegan’ packages. Permutational ANOVAs (PERMANOVAs) were performed with the function ‘adonis’ in the package ‘vegan’ as described in Desgarennes *et al*. (2014). The major microbial player analyses and graphics were performed using the average relative abundance and relative frequency of each OTU in each community (i.e. sample) across the three *Agave* species.

## Results

### Microbial community diversity associated with *Agave* species

We analyzed the prokaryotic and fungal communities associated with six sample types taken from three *Agave* species through Illumina iTag sequencing on the MiSeq platform. We obtained 35 770 987 and 27 596 665 total high‐quality reads (Fig. S1), which resulted in 3923 and 3173 OTUs after applying technical reproducibility thresholds for the prokaryotic and fungal data sets (Fig. S2), respectively. The majority of all prokaryotic and fungal OTUs discovered in the endospheres (leaf and root) in this analysis were also present in the episphere (rhizosphere and phyllosphere) and surrounding soils (Fig. 1d). Similarly, the majority of OTUs associated with the aboveground portions of the plant were also present in the belowground plant tissues and soil‐derived samples, although there was a considerably larger portion of fungal OTUs than prokaryotic OTUs (16.8 and 2.2%, respectively) detected solely in the aboveground tissues (Fig. S3). Intriguingly, the percentage of OTUs shared between sampling sites in Mexico and California was considerably smaller for fungal data sets than for the prokaryotic data sets (18.2 and 72.2%, respectively; Fig. S3), suggesting that fungal communities were perhaps more shaped by geographic distance. We also determined that *A. salmiana* shared a larger fraction of prokaryotic OTUs with the other native species, *A. deserti,* than with the other agave collected in Mexico, the cultivated *A. tequilana*, while the opposite trend was observed for fungal OTUs (Fig. 1e).

**Figure 2:**
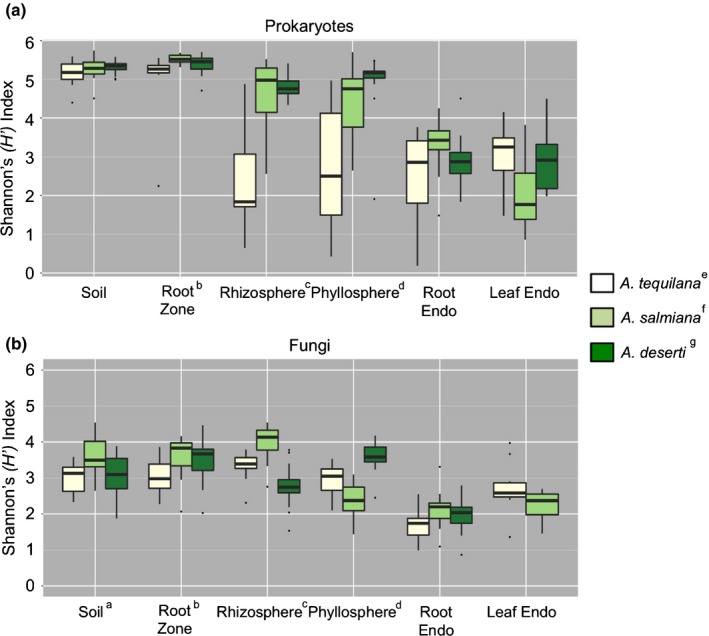
Estimated Shannon (*H’*) index in the (a) prokaryotic and (b) fungal communities associated with each sample type for the three *Agave* species, shown with ± SE. Superscripts (a–d) indicate significant differences in the marked plant compartment between plant species, while superscripts (e–g) indicate significant differences between sample types associated with a plant species. Statistical support is detailed in Supporting Information Table S1.

The levels of microbial diversity differed significantly among the sample types and across *Agave* species, except for the root and leaf endosphere and the prokaryotic bulk soil (Fig. 2a; Table S1). In general, alpha diversity as measured by the Shannon (*H’*) index decreased in value from soil to episphere to endosphere (Fig. 2a,b). Alpha diversity was marginally higher in the root zone soil than in the bulk soil for both prokaryotic and fungal communities across all three *Agave* species, suggesting that root exudates or other plant‐associated factors may be responsible for increasing local microbial diversity. Remarkably, the prokaryotic alpha diversity associated with both the rhizosphere and phyllosphere of the cultivated *A. tequilana* was substantially lower than that of the two wild agaves, suggesting a loss of natural microbial diversity (Fig. 2a; Table S1). Furthermore, these levels were even lower than the average values for the respective endophytic compartments of *A. tequilana*. However, it was surprising that microbial diversity in the phyllosphere for all three agaves was almost as high as diversity in the rhizosphere, despite the harsher environmental conditions that prevail for aboveground plant surfaces than for underground surfaces.

**Figure 3:**
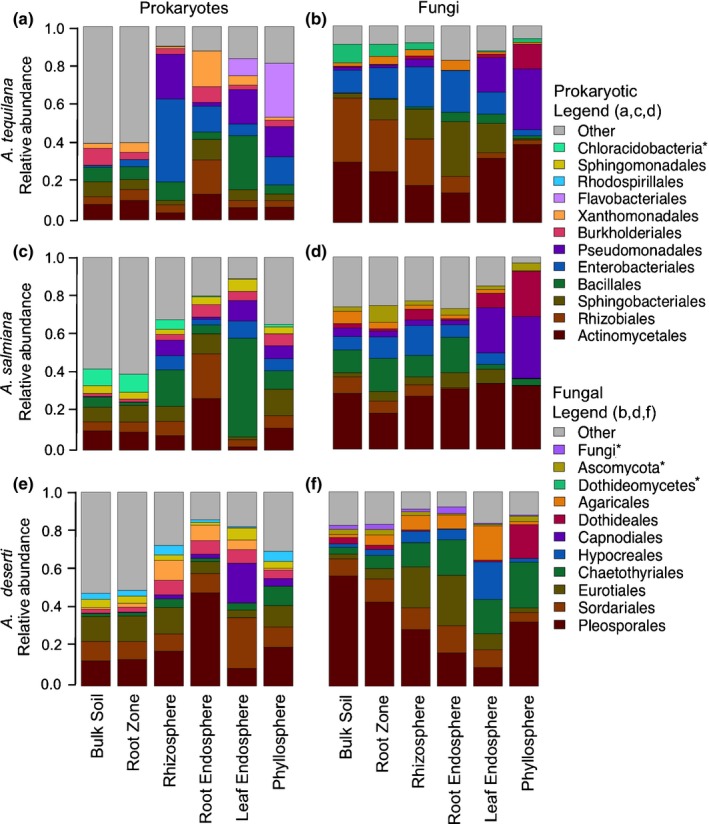
Order‐level relative abundance plots of prokaryotic (a, c, e) and fungal (b, d, f) communities by sample type for *A. tequilana* (a, b), *A. salmiana* (c, d), and *A. deserti* (e, f). Asterisks in the legend indicate taxonomic bins containing operational taxonomic units that could not be resolved to the order level.

Across all samples, we detected a total of 33 distinct prokaryotic phyla, although just four of these (*Proteobacteria*,* Actinobacteria*,* Firmicutes* and *Bacteroidetes*) comprised on average > 80% of each plant‐associated sample type (rhizosphere, root endosphere, leaf endosphere, and phyllosphere; Fig. S4). The plant‐associated sample types (epispheres and endospheres) were enriched for *Proteobacteria* (Kruskal–Wallis χ^2^ = 50.84; *P < *1.00 × 10^−12^) and *Actinobacteria* (Kruskal–Wallis χ^2^ = 11.07; *P < *8.790 × 10^−4^) and depleted for *Acidobacteria* (Kruskal–Wallis χ^2^ = 125.51; *P < *2.20 × 10^−16^) with respect to the surrounding soils, consistent with previous observations for *Agave* and other plants (Bulgarelli *et al*., 2012; Lundberg *et al*., 2012; Shakya *et al*., 2013; Desgarennes *et al*., 2014). While the prokaryotic relative abundance profile seemed to be similar in the bulk and root zone soil samples, community composition differed substantially between the rhizosphere, root endosphere, leaf endosphere, and phyllosphere (Fig. 3). By contrast, the transition in relative abundance profiles between sample types for fungal communities was more gradual than discrete (Fig. 3), with several lineages exhibiting proportional increases or decreases in abundance across bulk soil, root zone, rhizosphere and root endosphere compartments. Fungal communities across all six sample types were dominated by the phylum *Ascomycota* (average 91.2% of total relative abundance), while *Basidiomycota* represented a much smaller portion of the communities (7.7%) (Fig. S4). Strikingly, very few members of the known arbuscular mycorrhizal fungi (AMF) phylum *Glomeromycota* (36 OTUs; 1.1%) were detected in the soil, root zone soil, rhizosphere and root endosphere of the native agaves. Those that were detected were members of *Entrophospora* and *Glomus* spp*.,* and were predominantly associated with the two wild agaves (Kruskal–Wallis χ^2^ = 23.79; *P < *1.01 × 10^−6^). Mycorrhizal species were virtually absent from all samples from *A. tequilana*, including their associated soils.

We observed a number of differences in prokaryotic and fungal relative abundance patterns between the three *Agave* species. For example, in both *A. tequilana* and *A. deserti*, the fungal orders *Pleosporales* and *Eurotiales* decreased and increased in average relative abundance, respectively, across the transition from bulk soil to root endosphere (Fig. 3b,f). In *A. salmiana*, however, relative abundances of both these taxonomic groups were relatively stable between these sample types. By contrast, the fungal order *Capnodiales* represented a significant portion of the aerial microbiomes of both Mexican agaves (Fig. 3b,d), but was nearly absent from all sample types in *A. deserti* (Kruskal–Wallis χ^2^ = 28.64; *P < *8.70 × 10^−7^). Similarly, while the prokaryotic order *Bacillales* represented a substantial portion (> 20%) of the leaf endosphere of both Mexican agaves, it represented < 5% of the leaf endosphere community for *A. deserti* (Kruskal–Wallis χ^2^ = 12.65; *P < *3.77 × 10^−4^).

The rhizospheres of the cultivated *A. tequilana* were enriched for the bacterial orders *Pseudomonadales* (Kruskal–Wallis χ^2^ = 6.61; *P < *9.97 × 10^−3^) and *Enterobacteriales* (Kruskal–Wallis χ^2^ = 21.37; *P < *3.79 × 10^−6^) with respect to the native rhizospheres (Figs 3a, S5). Combined, the two orders comprised as much as 92% of a single *A. tequilana* rhizosphere sample, and on average represented 66% of the rhizosphere communities of the cultivated plants. By comparison, the rhizospheres of the wild *A. deserti* and *A. salmiana* showed considerably more diversity, with the most abundant orders *Actinomycetales* and *Bacillales* representing on average only 14 and 10%, respectively, of the native rhizosphere communities. The prokaryotic communities of the bulk soil were more similar across all sampling locations, and *Enterobacteriales* or *Pseudomonadales* comprised < 1% of total community abundance in all cases. Interestingly, the phyllosphere of the cultivated agave had high average levels of the bacterial orders *Pseudomonadales*,* Enterobacteriales* and also *Flavobacteriales* (Fig. 3a), which together comprised on average 58% of the phyllosphere community. The abundance of these few lineages in the epispheres of the cultivated *A. tequilana* came at the expense of many other depleted orders found to be significantly more abundant in the native rhizospheres, including *Pirellulales (*Kruskal–Wallis χ^2^ = 8.19; *P < *4.20 × 10^−3^), *Solibacterales* (Kruskal–Wallis χ^2^ = 8.23; *P < *4.05 × 10^−3^), *Acidimicrobiales* (Kruskal–Wallis χ^2^ = 12.85; *P < *3.39 × 10^−4^) and *Rhizobiales* (Kruskal–Wallis χ^2^ = 13.25; *P < *2.73 × 10^−4^).

### Factors driving microbial communities in *Agave* at the global scale

PERMANOVAs of both prokaryotic and fungal communities evaluating all the factors considered in the experimental design and their interactions revealed that sample type, the biogeography of the host plant species and the interaction of these two factors had the most influence on microbial communities, although in the prokaryotic communities sample type accounted for most of the variance (63%; Table 2), while in fungal communities the biogeography of the host plant species played the largest role (52.7% of variance; Table 2). Season and the interaction between sample type and season also had significant, albeit small, influences on the fungal communities, which were not observed at the global scale in the prokaryotic assemblages. It is worth remembering that in these global analyses the factor site could not be evaluated independently of plant species, as the *Agave* species used in this study do not grow in sympatry. PERMANOVA results also confirmed that variation among the three independent replicate samples of the same sampling time/condition in each species was generally low (small residuals in each *F* test; data not shown).

**Table 2:** PERMANOVA analyses of the microbial communities associated with *Agave* species considering all factors and their interactions (only significant factors are displayed; *P ≤* 0.05)

Prokaryotes				Fungi			
Factor^a^	*F*	*R* ^2^	*P*	Factor^a^	*F*	*R* ^2^	*P*
Global							
Sample type_5,199_	183.247	0.631	0.001	Species_2,190_	350.97	0.527	0.001
Species_2,199_	107.096	0.148	0.001	Sample type_5,190_	49.04	0.184	0.001
Sample type: species_10,199_	10.517	0.072	0.001	Sample type: species_10,190_	16.83	0.126	0.001
				Sample type: season_5,190_	1.94	0.007	0.016
				Season_1,190_	2.54	0.002	0.050
Bulk soil and root zone soil							
Species_2,71_	88.056	0.667	0.001	Species_2,71_	190.848	0.820	0.001
				Sample type_1,71_	4.284	0.009	0.019
Rhizosphere and phyllosphere							
Species_2,70_	88.016	0.591	0.001	Species_2,70_	254.033	0.709	0.001
Sample type_1,70_	32.056	0.108	0.001	Sample type: species_2,70_	34.259	0.096	0.001
Sample type: species_2,70_	7.142	0.048	0.001	Sample type_1,70_	56.940	0.080	0.001
				Season_1,70_	2.985	0.004	0.049
Root and leaf endosphere							
Sample type_1,58_	110.965	0.479	0.001	Sample type_1,49_	57.269	0.351	0.001
Species_2,58_	12.546	0.108	0.001	Species_2,49_	17.771	0.218	0.001
Sample type: species_2,58_	7.274	0.063	0.001	Sample type: species_2,49_	4.451	0.055	0.001
Species: season_1,58_	5.228	0.045	0.001	Season_1,49_	3.756	0.023	0.008
Season_2,58_	4.624	0.020	0.014	Sample type: season_1,49_	3.513	0.022	0.014
Sample type: season_1,58_	3.364	0.014	0.027	Sample type: species: season_1,49_	2.617	0.016	0.049

a Subscript numbers indicate the degrees of freedom and residuals of each *F* test.

**Figure 4:**
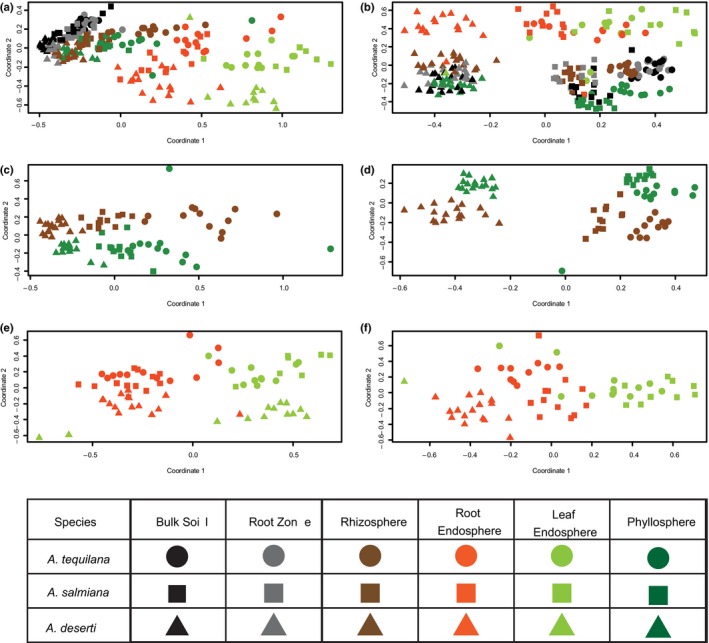
Nonmetric multidimensional scaling (NMDS) plots for Bray–Curtis distances of prokaryotic and fungal communities associated with *Agave* species. (a, b) All six sample types; (c, d) only rhizospheres and phyllospheres; (e, f) only root and leaf endospheres.

PERMANOVA results were corroborated by NMDS plots using the Bray–Curtis distance, where both the prokaryotic and fungal data sets displayed clustering by sample type, geography and host species, but the relative contributions of each factor differed across the two data sets (Fig. 4). Similar patterns of clustering were observable for the 16S data set using unweighted UniFrac distances (Fig. S6); weighted UniFrac distances exhibited less distinct clustering by any of the factors. NMDS ordination for the ITS data set was not performed with UniFrac because of known issues with generating accurate phylogenetic trees from the hypervariable ITS2 sequence data (Lindahl *et al*., 2013). In the case of the prokaryotic communities, the clusters that formed corresponded mainly to the different sample types, starting from the soils at one end (soil and root zone soil), followed by the rhizosphere and phyllosphere, and the root and leaf endospheres at the far end (Fig. 4a). The NMDS plots revealed that endosphere samples exhibited the greatest between‐sample variation (Fig. 4a,b), while soil samples clustered more closely together. Similarly, variation in levels of alpha diversity for endospheres in the prokaryotic communities was larger than for soil samples (Fig. 2a). The prokaryotic samples formed subclusters within each sample type based on the biogeography of each host species, confirming the PERMANOVA results shown in Table 2. In the case of the fungal communities, the global NMDS plot (Fig. 4b) revealed that there was a clear distinction between samples from California (left) and Mexico (right), and that soils clustered close to the rhizosphere and phyllosphere, while endophytic communities were grouped separately from them (Fig. 4b). As sample type was a major factor contributing to data clustering, we evaluated the impact of all factors in the epiphytic (Fig. 4c,d) and endophytic communities (Fig. 4e,f) separately. While both prokaryotic and fungal epiphytic data sets were mainly influenced by the biogeography of the *Agave* species, the influence of this factor was greater in the fungal than in the prokaryotic communities. This trend is visually supported by the NMDS and numerically reflected by the PERMANOVA results (Table 2). In the episphere, sample type and the interaction sample type–species explained together 15.6 and 17.6% of the variance in prokaryotes and fungi, respectively. Season played only a minor role in the epiphytic fungal communities (Table 2). Statistical analyses of the endosphere of agaves suggested that these prokaryotic and fungal communities were influenced not only by the sample type, plant species and their interaction (which accounted for 65 and 62.4% of the total variance in prokaryotes and fungi, respectively), but also by the factor season and the interaction of this factor with the others, which explained 7.9% of the variance in prokaryotes and 6.1% in fungi (Fig. 4e,f; Table 2). NMDS results also confirmed that the three independent samples taken at each sampling time/condition generally clustered close to each other, as depicted in Fig. S7(a–f), indicating that intrasample variation between replicates was generally low.

### Factors driving microbial communities in *Agave* at the species scale

We were also able to investigate the effect of all factors and their interactions, including the effect of geographic location, within each *Agave* species, as our experimental design considered at least two locations for each species (two 240 km apart for *A. tequilana*, two 130 km apart for *A. salmiana*, and three within 50 km for *A. deserti*; Table 1). From these analyses, in which we performed statistical tests similar to those described above (Fig. S5; Tables S1–S3), we are able to conclude that the microbial communities associated with the two native agaves behaved similarly for the factors evaluated. In both native species, sample type explained most of the variance across samples, although its influence was greater in the prokaryotic communities than in the fungal ones (85–81% in prokaryotes; 74–62% in fungi for *A. salmiana* and *A. deserti*, respectively). Site played a minor role in the prokaryotic communities of *A. salmiana* (2.2%; Table S2) and none in those of *A. deserti* (Table S3). Fungal communities of *A. deserti* were more influenced by the site and the interaction of site with the sample type (explaining together 15% of the variance), while in *A. salmiana* the factor site only accounted for 1.5% of the variance in the fungal data set. By contrast, in the cultivated agave the factors site and season did influence both prokaryotic and fungal communities (Table S1), suggesting that abiotic factors played a more substantial role in shaping the microbiome of *A. tequilana*.

### Major microbial players associated with *Agave* – the endosphere

Inspired by the possibility of approaching ecological relationships as microbial markets (Werner *et al*., 2012), we made use of the Pareto concept (the 80–20 rule) to identify those prokaryotic and fungal taxa accounting for 80% of *Agave*'s microbial communities. After their identification, we plotted their average relative abundance and frequency across each sample type in each *Agave* species to infer functional relationships and ecological relevance (Figs S8–S13).

**Figure 5:**
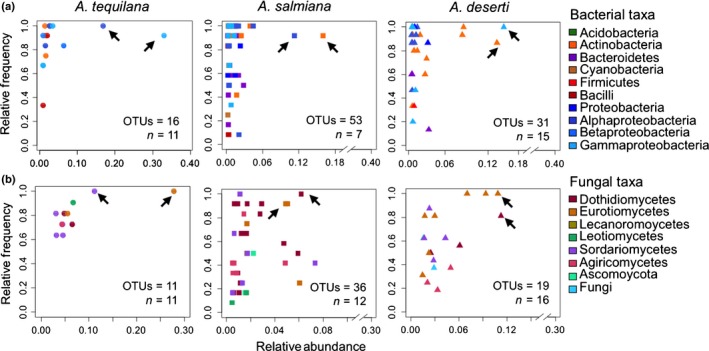
Relative frequency versus relative abundance of major operational taxonomic unit (OTU) players associated with the root endosphere of agaves for (a) prokaryotes and (b) fungi. Arrows indicate most abundant genera in each case. The number of OTUs and the number of samples (*n*) are indicated in the bottom right corner of each plot.

In general, our analyses confirmed low levels of microbial diversity associated with cultivated *A. tequilana*, as we identified a reduced number of microbial taxa playing a major role in this agave in comparison with the native species (Figs 5, S8, S11). Despite this reduction, our analyses also revealed that there were a number of shared major players at the phylum/class level across the three *Agave* species studied. At the taxonomic level of individual OTUs, we observed different microbes playing major roles between native and cultivated agaves.

**Figure 6:**
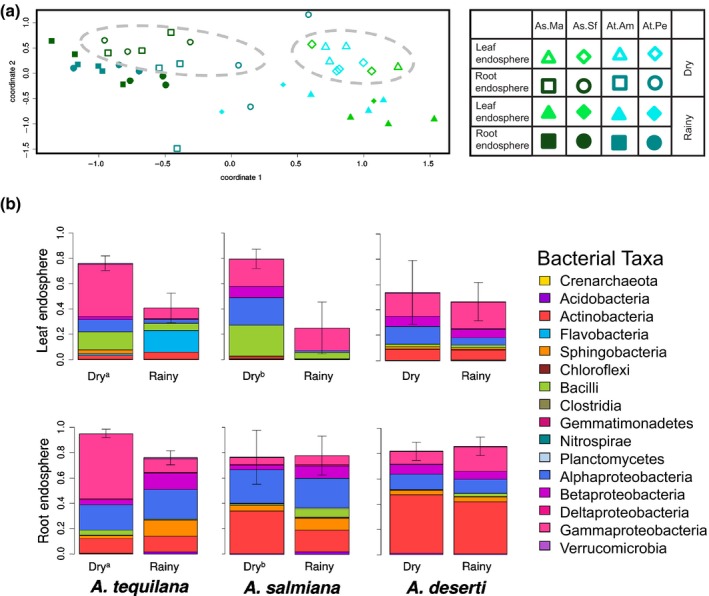
Endophytic core of agaves. (a) Nonmetric multidimensional scaling (NMDS) plot of the endophytic prokaryotic communities from *Agave tequilana* and *A. salmiana*; grey ellipses note dry season samples. As.Ma, *A. salmiana* from site El Magyuel; As.Sf, *A. salmiana* from San Felipe; At.Am, *A. tequilana* from Amatitan; At.Pe, *A. tequilana* from Penjamo. (b) Shared core bacterial operational taxonomic units (belonging to *Actinobacteria*,* Bacilli*, and *Alpha‐*,* Beta‐* and *Gammaproteobacteria*) in the root and leaf endosphere of agaves in the dry and rainy seasons. Kruskal–Wallis test (leaf: χ^2^ = 7.5; df = 1; *P*‐value = 0.00617 for *A. tequilana*; χ^2^ = 4.5; df = 1; *P*‐value = 0.03389 for *A. salmiana;* root: χ^2^ = 8.31; df = 1, *P*‐value = 0.0039 for *A. tequilana*). Relative abundance profiles of the core prokaryotic taxa, indicated in the legend ± SD for the total relative abundance, are displayed.

We found that *A. tequilana*'s prokaryotic leaf endosphere was dominated by OTUs identified as *Acinetobacter* and *Bacillus* (12% each), while *Leclercia* (8–30%) was the dominant OTU for this compartment in the native agaves (Figs S8–S10). We observed that in *A. tequilana*'s root endosphere the major players were OTUs identified as *Stenotrophomonas* (33%) and *Agrobacterium* (17%) (both *Proteobacteria*). By contrast, the root endosphere of native agaves was dominated by *Actinosynnemataceae* and *Promicromonospora* (16% for each, both belonging to *Actinobacteria*), plus *Rhizobiales* (11%) in *A. salmiana* and *Leclercia* (15%) in *A. deserti* (Fig. 6a). Notably, the differences observed in the major bacterial players in the endophytic compartment were not evident in the soil communities across the three plant species (Figs S8–S10).

In the fungal communities, we found that in the leaf endosphere of *A. tequilana* and *A. salmiana* three of the most abundant OTUs belonged to *Alternaria* (9 and 14%, respectively), *Penicillium* (7% in *A. tequilana*) and *Cladosporium* (20% in *A. salmiana*) (Figs S11, S13). By contrast, in *A. deserti’*s leaf endosphere the dominant OTUs were identified as *Psathyrella* (17%) and *Cyphellophora* (13%) (Fig. S13). Likewise, we observed that native agaves’ root endospheres were dominated by *Lophiostoma* (6–11%), plus *Cladosporium* (6% in *A. salmiana*) and *Cyphellophora* (11% in *A. deserti*), while *A. tequilana*'s root endosphere was dominated by *Penicillium* (28%) and *Fusarium* (11%) (Fig. 5b). Finally, we observed that the number of fungal OTUs and taxa detected in the proximal and distal soil communities in the native agaves was greater than those observed in *A. tequilana* (Figs S9–S11).

### Is there a dry core endophytic microbiome in *Agave*?

Our experimental design allowed us to evaluate the impact of naturally occurring dry periods on the microbial communities associated with *Agave*. Our *Agave* samples from Mexico were collected at the highest peak of drought (dry season) and after 4 months of variable levels of precipitation (rainy season) (Table 1). Based on results from NMDS and PERMANOVA, which suggested that season influenced prokaryotic and fungal endophytic communities (Fig. 3e,f; Table 2), we investigated whether root and leaf endophytic major microbial players were affected by these seasonal changes (Fig. S14; Tables S4, S5). In the case of the endophytic prokaryotic communities, we observed that a core group formed by OTUs of *Actinobacteria*,* Bacilli*,* Alpha‐*,* Beta‐* and *Gammaproteobacteria* was enriched during the dry season in comparison to the rainy season for leaf endospheres of *A. tequilana* and *A. salmiana*, as well as for the root endosphere of *A. tequilana*, but not in either endosphere of *A. deserti* (Figs 6a,b, S14; Tables S4, S5). This may be explained by the fact that *A. deserti* occupies the driest habitat of the three species examined (Nobel, 2003), experiencing smaller seasonal variations.

In contrast to these differences observed for prokaryotic communities, fungal endophytic communities were less affected by season (Fig. S15; Table S5). At higher taxonomic levels, the leaf endospheres associated with *A. tequilana* and *A. salmiana* were similar to one another across both dry and rainy seasons (Fig. S15). In general, while fungal root endosphere communities of the three *Agave* species could be differentiated from one another (Fig. 4f) based on total relative abundance patterns, they did in fact share a core of eighty OTUs representing *c*. 40% or more of the total community in each species (Figs S14, S15).

## Discussion

### Distinct factors shape prokaryotic and fungal communities in *Agave*

The microbial communities associated with plant hosts are probably shaped by a wide variety of environmental and host‐related factors, including geographic location, plant phenotype and genotype, soil chemistry, and seasonal effects. This study determined that prokaryotic communities associated with agaves were chiefly influenced by the sample type or plant compartment, where the rhizosphere, the phyllosphere and the root and leaf endosphere were clearly distinct from one another and also from the surrounding soils. These results were previously suggested for agaves (Desgarennes *et al*., 2014), but by using next‐generation sequencing (NGS), these differences were made more clear even among epiphytic groups. Moreover, by including a Californian species, *A. deserti*, living > 2000 km apart from *A. tequilana* and *A. salmiana*, our data revealed the convergence of prokaryotic communities in *Agave*, regardless of cultivation status and biogeography. Interestingly, fungal communities presented a different pattern, in which the biogeography of the plant host species played the dominant role. Recent studies in several different experimental systems have similarly reported that fungal communities appear to be differentiated more by geographic distance than are prokaryotic communities (Peay *et al*., 2007; Shakya *et al*., 2013; Meiser *et al*., 2014), which suggests that fungal endemism may be a community‐shaping force operating at multiple scales and in multiple habitats. One hypothesis suggests that dispersal limitation is the root cause of this phenomenon (Taylor *et al*., 2006), and that fungi behave more like plants and animals than do bacteria in this respect. Our analyses and comparisons across sampling sites within individual species substantiate the suggestion that geography plays a larger role in driving fungal than prokaryotic communities. The considerably smaller proportion of shared fungal OTUs than prokaryotic OTUs between samples collected in California and Mexico lends further support to this hypothesis. Nevertheless, the six microhabitats or sample types investigated represent, in both prokaryotic and fungal data sets, a source of major selection. Prokaryotic and fungal epiphytic communities in *Agave* revealed a strong influence of the plant species’ biogeography (Fig. 4c,d), which can be related to a plant selection and/or niche effect as previously discussed (Desgarennes *et al*., 2014; Mendes *et al*., 2014). To what extent this phenomenon depends not only on the biogeography of the plant host species, but also on the host genotype must still be investigated by comparing microbial communities of agaves with those associated with other sympatric *Agave* or non‐*Agave* species in these arid environments. Recent studies have demonstrated that plant host‐specific traits, including broad morphological characteristics (Kembel *et al*., 2011) and specific genetic pathways and gene products (Horton *et al*., 2014; Lebeis *et al*., 2015), can have significant effects on microbiome composition and diversity. In the endosphere, an influence of the season was observed in the Mexican agaves (Figs 6, S13; Table S4), especially in the prokaryotic communities, as detected previously (Desgarennes *et al*., 2014). Leaf endophytic fungal communities were similar in *A. tequilana* and *A. salmiana*, which suggests common mechanisms of plant–fungus interaction in this habitat.

### Low levels of microbial diversity in the cultivated *A. tequilana* compared with native agaves

Cultivated agaves are susceptible to a number of diseases, many of which cause significant losses in yield and revenue each year (Dalton, 2005). One of the most costly of these diseases, called ‘soft rot’, was previously estimated to have cost the tequila industry $200 million yr^−1^ (Jiménez‐Hidalgo *et al*., 2004). A putative causative agent of ‘soft rot’ has been identified as a member of the bacterial family *Enterobacteriaceae* (*Pantoea agglomerans*) (Jiménez‐Hidalgo *et al*., 2004). In this study, we observed significantly lower levels of prokaryotic diversity in the rhizosphere and phyllosphere of the cultivated *A. tequilana* compared with the native *A. salmiana* and *A. deserti*. Furthermore, this lower alpha diversity was the direct result of the dominance in the epispheres of *A. tequilana* of a few bacterial families, including genera belonging to *Enterobacteriaceae* (*Pantoea*,* Leclercia*, and *Trabusiella*). The lack of enrichment for *Enterobacteriaceae* in the surrounding bulk soils suggests that these bacterial species have adapted to specific associations with their plant host. A number of agronomic and regulatory practices within the tequila industry may be potentially influencing prokaryotic alpha diversity in *A. tequilana*. First, continuous monocultures, as is typically the case on agave plantations, have been shown to decrease microbial community diversity (Hilton *et al*., 2013). Second, tequila plantations typically grow young agave plants indoors for a period of time before transplanting to the field, and, before transplant, the roots are sterilized in formaldehyde and left to dry for a period of time (Davis *et al*., 2011). As rhizosphere microbial communities have been shown to assemble very early in plant development (Chaparro *et al*., 2014), root sterilization probably alters the progression and development of a natural microbial community. Third, governmental regulations that limit the available genetic pool and propagation techniques (vegetative reproduction via bulbils and rhizome offshoots preferred) result in agave plantlets that are genetically identical to the parental line (Valenzuela, 2011). High genetic homogeneity of the *A*. *tequilana* cultivar used in the tequila industry may have allowed the pathogenic microbes to evolve strategies to evade the limited arsenal of available host defenses. Current research aimed at adapting agave as a biofuels feedstock is exploring the use of a variety of *Agave* species, including the main cultivars from both the fiber and alcohol industries as well as traditionally uncultivated species (Garcia‐Moya *et al*., 2011; Escamilla‐Treviño, 2012; Li *et al*., 2014), thereby potentially increasing the cultivated agave's resilience to pathogen attack by introducing additional defensive capabilities into the collective gene pool. Moreover, vegetative reproduction of *Agave* species either in agricultural settings or in natural environments may also have an influence on the endophytic communities, as microorganisms residing inside the mother plant could be potentially inherited by the offshoots and remain present in internal tissues despite surface sterilization. Our observation that endosphere samples exhibited greater between‐sample variability, compared with episphere and soil samples, could be explained in part by a combination of the stochastic nature of endosphere colonization and this cross‐generational propagation of endophytic microbes. Further research is required to evaluate the heritability of the plant microbiome both in the episphere and in the endosphere compartments of agaves.

### Major microbial players highlight convergence across the genus *Agave*

In accordance with our global NMDS and PERMANOVA analyses, an investigation of the major microbial players demonstrated that the prokaryotic and fungal epiphytic and endophytic communities had a reduced number of OTUs playing a major role in cultivated *A. tequilana* in comparison with the native species *A. salmiana* and *A. deserti*. This analysis also revealed that a group of prokaryotic and fungal taxa was conserved across the three *Agave* species, although the relative distribution of the common taxa varied across the three hosts. This result is consistent with findings from recent studies in which the microbiomes of closely related species or cultivars exhibit both specific microbial lineages with host‐specific abundance patterns and a conserved core microbiome (Schlaeppi *et al*., 2014; Bulgarelli *et al*., 2015; Haney *et al*., 2015; Lebeis *et al*., 2015). The variation we observed can be explained by the fact that these agaves are native to different habitats and represent different genotypes, as microbial communities are assembled on niche‐based processes, as a result of the plant selection effect and environmental factors (Mendes *et al*., 2013). Interestingly, we found that differences in bulk soil prokaryotic communities seemed not to be significantly correlated with species management status. By contrast, we found a reduced number of major fungal players associated with *A. tequilana* soils in comparison with those of native agaves.

Interestingly, prokaryotic communities in the desert environments surveyed here do not differ markedly at the phylum level from other soil environments described previously in other phytobiome studies (Lundberg *et al*., 2012; Bulgarelli *et al*., 2015; Edwards *et al*., 2015; Zarraonaindia *et al*., 2015). Similarly, the increased abundance of *Proteobacteria* and decreased presence of *Acidobacteria* in the plant‐associated samples with respect to the surrounding soil has been observed for other plant hosts (Edwards *et al*., 2015; Zarraonaindia *et al*., 2015). Taken together, these findings suggest the presence of a set of conserved forces acting to shape the structure of both the plant‐associated and soil prokaryotic microbiomes across a wide array of environments and host species. For fungi, however, most temperate forests are dominated by *Basidiomycota* (O'Brien *et al*., 2005); the fungal communities associated with agaves described here were dominated by members of *Ascomycota*. This is consistent with studies of other semiarid plants such as perennial grasses, which are also predominantly colonized by *Ascomycota* (Porras‐Alfaro *et al.,* 2011), and is also consistent with the trend we observed within our study that differences in fungal communities are correlated with differences in geographic distance.

The presence of *Glomeromycotan* sequences in our study was low, but we detected a preferential association of the *Entrophospora* genus with *A. salmiana* and *Glomus* with *A. deserti*. This apparent lack of *Glomeromycota* may in part be attributable to primer bias, as it is well known that ‘universal fungal’ primers often fail to amplify these basal organisms (Martin & Rygiewicz, 2005). Ongoing metagenomic studies on the rhizosphere of agaves will help elucidate whether members of the *Glomeromycota* were underrepresented in this study.

### The endophytic core of *Agave*

Our extensive analyses on the *Agave*‐associated microbial communities suggested that the endospheres were less influenced by the biogeography of the host species, but more by the plant compartment and seasonal changes. We corroborated that indeed there is a group of prokaryotic organisms living inside agaves which increased in abundance under natural drought conditions, as already suggested (Desgarennes *et al*., 2014). We discovered that fungal leaf endophytic communities were similar across the Mexican agaves, and that these fungal taxa were not affected by season. Importantly, a broader group of taxa were shared across *Agave* species in the root endosphere, representing in all cases > 40% of the community. As previously mentioned, *Agave* species reproduce in nature mostly through vegetative offshoots that remain connected to the mother through their lives (Gentry, 1982), probably enhancing the heritability of the endophytic microbial symbionts. As recent studies have shown that plant microbial communities can change significantly across the life span of a single plant for even annual species (Copeland *et al*., 2015), it will be interesting to investigate successional dynamics in perennial species with asexually reproducing lifestyles. Some of the microorganisms shared among the three *Agave* species, among them several previously reported diazotrophic strains (Desgarennes *et al*., 2014), have been isolated in our laboratories and will be sequenced in the near future. These efforts will provide a genomic baseline to further deepen our understanding of the complex plant–microbe interactions in arid and semiarid ecosystems.

## Author contributions

S.G., D.C‐D., G.N., T.W., A.V., L.P.P‐M. and S.G.T. planned and designed research; D.C‐D., D.D., C.F‐G. and S.G. performed experiments; S.G., G.N., D.D., C.F‐G. and L.P.P‐M. conducted fieldwork; D.C‐D. and S.C. prepared libraries and processed sequencing data; D.C‐D., D.D., C.F‐G. and L.P.P‐M. analyzed the data; and D.C‐D., D.D., A.V., L.P.P‐M. and S.G.T. wrote the manuscript. All authors read and approved the final manuscript.

## Supporting information

Please note: Wiley Blackwell are not responsible for the content or functionality of any supporting information supplied by the authors. Any queries (other than missing material) should be directed to the *New Phytologist* Central Office.

**Fig. S1** Raw read counts across all samples grouped by sample type.
**Fig. S2** Established cut‐off thresholds for technical reproducibility across OTUs for 16S and ITS2 data sets.
**Fig. S3** OTU distributions across sample subsets.
**Fig. S4** Phylum‐level relative abundance plots of prokaryotic and fungal communities associated with each *Agave* species by sample type.
**Fig. S5** Order‐level relative abundance plots of prokaryotic communities associated with all rhizosphere and bulk soil samples.
**Fig. S6** NMDS clustering for prokaryotic data of all samples using unweighted and weighted UniFrac distances.
**Fig. S7** NMDS clustering of all samples by sample type, host species and location according to the legend.
**Fig. S8** Major prokaryotic taxa associated with *Agave tequilana* based on the 80–20 rule (Pareto).
**Fig. S9** Major prokaryotic taxa associated with *Agave salmiana* based on the 80–20 rule (Pareto).
**Fig. S10** Major prokaryotic taxa associated with *Agave deserti* based on the 80–20 rule (Pareto).
**Fig. S11** Major fungal taxa associated with *Agave tequilana* based on the 80–20 rule (Pareto).
**Fig. S12** Major fungal taxa associated with *Agave salmiana* based on the 80–20 rule (Pareto).
**Fig. S13** Major fungal taxa associated with *Agave deserti* based on the 80–20 rule (Pareto).
**Fig. S14** OTU distributions across *Agave* species in the endosphere.
**Fig. S15** Fungal taxa associated with the endosphere of all three *Agave* species and their relative abundance at the dry and rainy seasons.
**Table S1** Estimated Shannon diversity in the prokaryotic and fungal communities associated with each *Agave* species
**Table S2** PERMANOVA of the microbial communities associated with *Agave tequilana* considering all factors and their interactions
**Table S3** PERMANOVA of the microbial communities associated with *Agave salmiana* considering all factors and their interactions
**Table S4** PERMANOVA of the microbial communities associated with *Agave deserti* considering all factors and their interactions
**Table S5** PERMANOVA of the microbial communities associated with the endosphere of *Agave tequilana* and *A. salmiana* considering all factors and their interactions
**Methods S1** Methods for sample collection, DNA extraction, PCR amplification, sequencing, and data processing, including statistical analyses.
