# Supplementary material for: Plant compartment and biogeography affect microbiome composition in cultivated and native Agave species
Source: New Phytol. 2015 Oct 15;209(2):798–811. doi: 10.1111/nph.13697 (PMC5057366; doi:10.1111/nph.13697)
Supplement: Supplementary file 1 — Fig. S1 Raw read counts across all samples grouped by sample type. Fig. S2 Established cut‐off thresholds for technical reproducibility across OTUs for 16S and ITS2 data sets. Fig. S3 OTU distributions across sample subsets. Fig. S4 Phylum‐level relative abundance plots of prokaryotic and fungal communities associated with each Agave species by sample type. Fig. S5 Order‐level relative abundance plots of prokaryotic communities associated with all rhizosphere and bulk soil samples. Fig. S6 NMDS clustering for prokaryotic data of all samples using unweighted and weighted UniFrac distances. Fig. S7 NMDS clustering of all samples by sample type, host species and location according to the legend. Fig. S8 Major prokaryotic taxa associated with Agave tequilana based on the 80–20 rule (Pareto). Fig. S9 Major prokaryotic taxa associated with Agave salmiana based on the 80–20 rule (Pareto). Fig. S10 Major prokaryotic taxa associated with Agave deserti based on the 80–20 rule (Pareto). Fig. S11 Major fungal taxa associated with Agave tequilana based on the 80–20 rule (Pareto). Fig. S12 Major fungal taxa associated with Agave salmiana based on the 80–20 rule (Pareto). Fig. S13 Major fungal taxa associated with Agave deserti based on the 80–20 rule (Pareto). Fig. S14 OTU distributions across Agave species in the endosphere. Fig. S15 Fungal taxa associated with the endosphere of all three Agave species and their relative abundance at the dry and rainy seasons. Table S1 Estimated Shannon diversity in the prokaryotic and fungal communities associated with each Agave species Table S2 PERMANOVA of the microbial communities associated with Agave tequilana considering all factors and their interactions Table S3 PERMANOVA of the microbial communities associated with Agave salmiana considering all factors and their interactions Table S4 PERMANOVA of the microbial communities associated with Agave deserti considering all factors and their interactions Table S5 PERMANOVA of the microbial [file NPH-209-798-s001.pdf]

***New Phytologist* Supporting Information**

Article title: Plant compartment and biogeography affect microbiome composition in cultivated and native *Agave* species

Authors: Devin Coleman-Derr, Damaris Desgarennes, Citlali Fonseca-Garcia, Stephen Gross, Scott Clingenpeel, Tanja Woyke, Gretchen North, Axel Visel, Laila P. Partida-Martinez, and Susannah Tringe

Article acceptance date: 31 August 2015

The following Supporting Information is available for this article:

Methods S1

Sample Collection. The rhizosphere is conventionally defined as the 2 mm of soil adhering to the root surface, while the rhizoplane is defined as the surface of the roots; the extreme dryness and sandy quality of the soils in which the *Agave* specimens were harvested meant in most cases that very little soil adhered to the roots upon their removal from the ground. To account for this, samples were harvested in the following manner. Bulk soil samples were collected by taking ~50 cm^3 of top soil located approximately 1m from each plant replicate. An additional soil sample referred to as 'root zone' soil was collected by taking ~ 50 cm^3 from underneath the *Agave* plant, within 10 cm of the plant root. Both bulk and root zone soil samples were stored in sterile Ziploc bags, transported to the lab, and stored at -80° Celsius. For each plant replicate, the following were also collected using sterile technique: ~30 gms of *Agave* root, and 20cm of *Agave* leaf (in each case, the third leaf from the inner whorl was harvested from healthy hosts with no obvious signs of disease or damage). Plant tissues were stored in ziploc bags and transported to the lab on ice. Rhizosphere/rhizoplane and phyllosphere samples were collected by adding ~50ml of sterilized epiphyte buffer (50mMKH_2PO_4, 50mM K_2HPO_4, 0.1%Triton X-100) to the Ziploc containing either root or leaf tissue, followed by mixing the sample and buffer on a tabletop shaker for 15 minutes. After mixing, the 50ml of rhizosphere and phyllosphere samples were transferred to 50ml conical tubes and flash frozen in liquid nitrogen and stored at -80° Celsius until DNA extraction. For root and leaf endosphere samples, the root and leaf tissues used in the previous step were first surface sterilized with sterilization buffer (1:20 dilution commercial bleach) for 15 minutes while mixing on a table top shaker. After surface sterilization, root and leaf tissue were dissected to 1m thin sections with a sterile blade and added to a 15% glycerol stock in 50 ml conical tubes; these tubes were stored at 4° Celsius for 2hrs to allow the cells to absorb the glycerol stock, and then stored at -80° Celsius.

DNA Extraction. DNA extractions for bulk soil and root zone samples were done with the MoBio™ PowerSoil DNA Isolation Kit (MoBio Laboratories, Carlsbad, CA, USA) (SDS/mechanical lysis) because of its ability to remove PCR inhibitors from the soil samples. Rhizosphere samples were first thawed on ice, pelleted at 4,000 rpm for 20 minutes at 4° Celsius, and prepped using the PowerSoil kit. Pellets ranged in size from 30 to 80 mg. Leaf episphere samples were thawed on ice and prepared using the MoBio™ PowerWater DNA Isolation Kit. For root and leaf endosphere samples, 100 mg of each frozen sample was ground to a powder in liquid nitrogen with a sterile mortar and pestle and was then prepped following a CTAB genomic DNA extraction protocol. In brief, frozen ground tissue was added to 1ml CTAB buffer (100mM Tris pH8, 20mM EDTA, 1.4 M NaCl, 2% PVP 40, 2% Hexadecyltrimethylammonium bromide, 0.2% Beta mercaptoethanol), and incubated at 65°

Celsius for I hour. Samples were centrifuged at 10,000g for 10 minutes at 4° Celsius, and the supernatant was added to 1mL chloroform/Isoamyl alchohol (24:1), centrifuged, and the gDNA was precipitated with isopropanol. DNA quantity was assessed with the Qubit Broad-range Assay kit (Life Technologies, Grand Island, NY, USA).

PCR and Sequencing. For Fungal ITS2 amplification, we used the ITS9F (5' - GAACGCAGCRAAIIGYGA- 3') and ITS4R (5' –TCCTCCGCTTATTGATATGC- 3') primer sets. For 16S rRNA v4 amplification, we used the established primer pair 515F (5' – GTGCCAGCMGCCGCGGTAA- 3') and 816R (5' GGACTACHVGGGTWTCTAAT- 3') primer set, along with PNA clamps to reduce chloroplast and mitochondrial contamination as in (Lundberg *et al.*, 2013). The use of PNAs in the 16S v4 amplification substantially reduced overall chloroplast and mitochondrial contamination and increased the number of prokaryotic reads for the root endosphere, leaf endosphere, and phyllosphere samples respectively. The amplification reactions were conducted in 96-well plate format, and for each sample were performed in triplicate with ~10ng template per reaction. The PCR conditions used were 94 degrees C for 3 min, followed by 30 cycles of 94 degrees for 45s, 78 degrees for 10s, 50 degrees for 60s, and 72 degrees for 90s, and finally by 72 degrees for 10 min then cooling to 4 degrees. Four negative controls and several technical replicates were included in each 96-well plate to measure levels of contamination and to determine OTU measurability thresholds (see Fig. S2). Triplicate reactions for each sample were pooled, and quantification was carried out with the Qubit High Sensitivity Assay kit (Life Technologies) on a Turner Biosystems fluorescence plate reader (Promega, Madison, WI, USA). Sets of 96 barcoded PCR products were pooled in equimolar ratios and cleaned up using the AMPureXP magnetic beads (Beckman-Coulter, Indianapolis, IN, USA). Paired-end 2 x 250bp sequencing of the barcoded amplicons was performed on a MiSeq machine running v2 chemistry (Illumina Inc, San Diego, CA, USA).

Data processing. The raw Fastq reads were processed using a custom pipeline developed at the Joint Genome Institute. In brief, after filtering 33,323,114 ITS2 and 39,624,119 16S raw reads for known contaminants (Illumina adapter sequence) and PhiX sequencing control, primer sequences were trimmed from the 5' ends of reads. Low-quality bases were trimmed from the 3' ends prior to read1 and read2 assembly with either Pandaseq (Masella *et al.*, 2012) for ITS2 data or FLASH (Magoč & Salzberg, 2011) for 16S data. The remaining 27,596,665 fungal and 35,770,987 prokaryotic high-quality merged reads (Fig. S1) were clustered into 25,871 and 40,759 operational taxonomic units (OTUs), respectively, at 95% and 97% identity using the UPARSE pipeline (Edgar 2013). Taxonomies were assigned to each OTU using the RDP Naïve Bayesian Classifier (Wang *et al.*, 2007) with custom reference databases. For the16S V4 data, this database was compiled from the May 2013 version of the GreenGenes 16S database (DeSantis *et al.*, 2006), the Silva 16S database (Quast *et al.*, 2013) and additional manually-curated 16S sequences, trimmed to the V4 region. For the ITS2 data, this database was built from the UNITE database (Koljalg, 2013). After taxonomies were assigned to each OTU, we discarded 1) all OTUs that were not assigned a Kingdom level RDP classification score of at least 0.5, 2) all OTUs that were not assigned to Kingdom Bacteria or Archaea for the 16S datasets, and 3) all OTUs that were not assigned to Kingdom Fungi for ITS2 datasets. Using several technical replicates included on each sequenced 96-well plate, we calculated a threshold for determining technical reproducibility for these OTUs using the progressive drop-out analysis described in Lundberg et al 2013 (Fig. S2). This threshold determines the minimum cumulative read count across all plates for an OTU to be included in the analysis, and was determined to be at least 7 reads in at least 5 samples for the 16S dataset and at least 2 reads in at least 5 samples for the ITS2 dataset, which yielded 4,395 and 4,237 high-abundance OTUs (respectively) for downstream analyses. To account for differences in sequencing read depth across samples, and in particular to deal with the low read counts obtained from leaf endosphere samples, all samples were rarefied to 1000 reads per sample; OTUs which were

reduced to less than one read per OTU after rarefaction were discarded to yield 3,173 fungal and 3,923 prokaryotic measureable, rarefied OTUs for downstream analysis.

Statistical analyses.

Microbial Taxa Enrichment
Statistical support for prokaryotic and fungal enrichment was derived from Kruskall-Wallis tests performed with the function "kruskall.test" in the R base package using measureable, rarefied abundance data.

Distance and dissimilarity matrices.
Bray-Curtis dissimilarities were calculated using the "vegdist" function of the R package *vegan* (Oksanen *et al.*, 2013) using measureable OUT abundances. UniFrac distances were generated using the "UniFrac" function in the R package *Phyloseq* (McMurdie and Holmes, 2013) using Newick format trees created with FastTree on MAFFT generated alignments of the measurable OTU sequences.

NMDS analyses
NMDS was performed using "Mass" and "vegan" packages as described in Desgarennes *et al.,* 2014. Measurable datasets were used for these analyses.

Diversity analyses
We calculated the Shannon Diversity (H´) using the package "BiodiversityR" in R. These values were calculated from the taxonomic abundance matrices generated from all measurable, rarefied OTUs.

PERMANOVA
Permutational multivariate analysis of variance (PERMANOVA) was performed with the function "adonis" in the R package *vegan* (Oksanen *et al.*, 2013), using the Bray-Curtis dissimilarities. The null hypotheses that no difference in microbial community composition exists across each of the experimental factors was tested (Anderson & Walsh, 2013). The significance threshold for PERMANOVA was set at $p < 0.001$, and was determined by permutational testing (1,000 and 10,000 permutations respectively). Measurable datasets were used for these analyses.

References

Anderson MJ, Walsh DCI. (2013). PERMANOVA, ANOSIM, and the Mantel test in the face of heterogeneous dispersions: What null hypothesis are you testing? *Ecol Monogr* **83**:557–574.

DeSantis TZ, Hugenholtz P, Larsen N, Rojas M, Brodie EL, Keller K, *et al.* (2006). Greengenes, a Chimera-Checked 16S rRNA Gene Database and Workbench Compatible with ARB. *Appl Environ Microbiol* **72**:5069–5072.

Desgarennes D, Garrido E, Torres-Gomez MJ, Peña-Cabriales JJ, Partida-Martinez LP. 2014. Diazotrophic potential among bacterial communities associated with wild and cultivated *Agave* species. FEMS Microbiology Ecology 90: 844–857.

Edgar RC. 2013. UPARSE: highly accurate OTU sequences from microbial amplicon reads. Nature Methods 10: 996–998.

Koljalg U. (2013). Towards a unified paradigm for sequence-based identification of fungi. *Mol Ecol* **22**:5271–5277.

Lundberg DS, Yourstone S, Mieczkowski P, Jones CD, Dangl JL. (2013). Practical innovations for high-throughput amplicon sequencing. *Nat Methods* **10**:999–1002.

Magoč T, Salzberg SL. (2011). FLASH: Fast Length Adjustment of Short Reads to Improve Genome Assemblies. *Bioinformatics*. doi: 10.1093/bioinformatics/btr507

Masella AP, Bartram AK, Truszkowski JM, Brown DG, Neufeld JD. (2012). PANDAseq: paired-end assembler for illumina sequences. *BMC Bioinformatics* **13**:31.

McMurdie PJ, Holmes S. (2013). phyloseq: An R Package for Reproducible Interactive Analysis and Graphics of Microbiome Census Data. *PLoS ONE* **8**:e61217.

Oksanen J, Blanchet FG, Kindt R, Legendre P, Minchin PR, O'Hara RB, *et al.* (2013). vegan: Community Ecology Package. http://CRAN.R-project.org/package=vegan. v.2.3.0.

Quast C, Pruesse E, Yilmaz P, Gerken J, Schweer T, Yarza P, *et al.* (2013). The SILVA ribosomal RNA gene database project: improved data processing and web-based tools. *Nucleic Acids Res* **41**:D590–D596.

Wang Q, Garrity GM, Tiedje JM, Cole JR. (2007). Naïve Bayesian Classifier for Rapid Assignment of rRNA Sequences into the New Bacterial Taxonomy. *Appl Environ Microbiol* **73**:5261–5267.

Figure S1

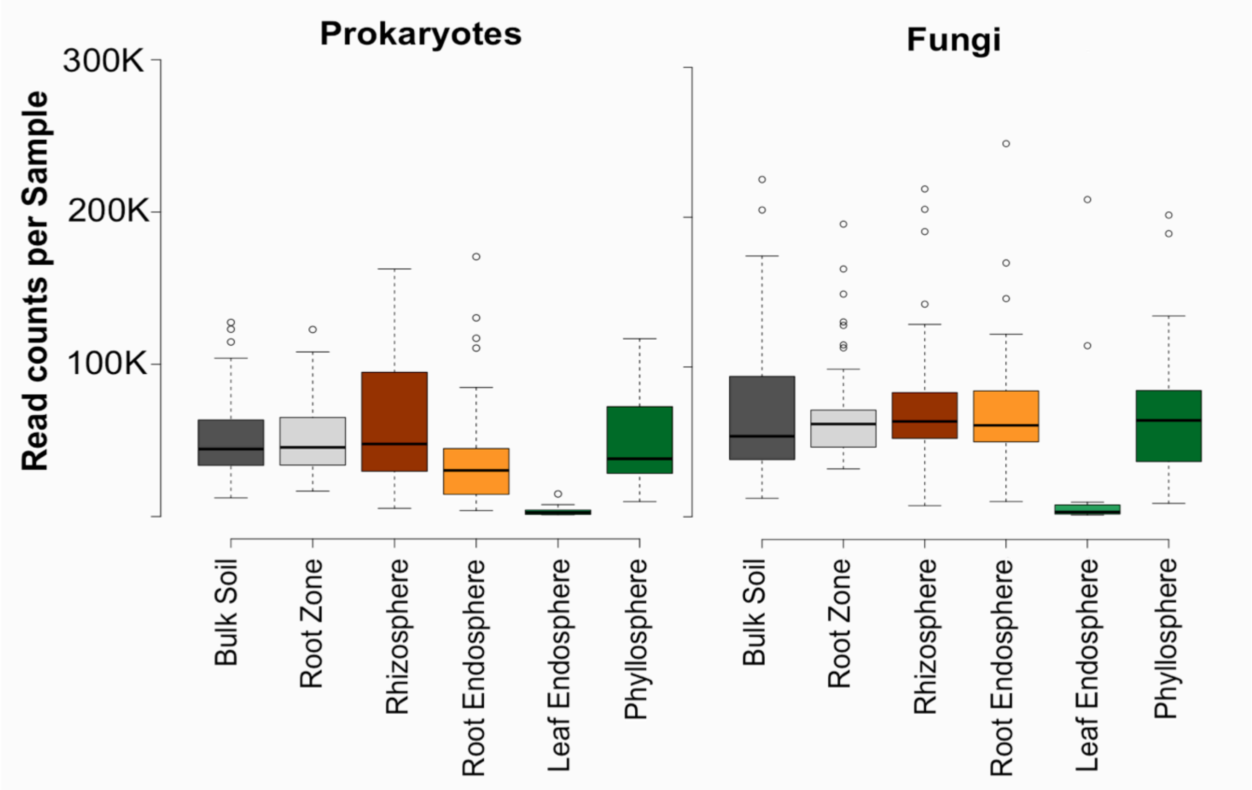

Figure S1. Raw read counts across all samples grouped by sample type. Boxplots of raw read counts per sample for the six sample types for prokaryote (left panel) and fungal (right panel) datasets. Read counts represented here are taken after removal of non-target sequences (chloroplast, mitochondrial, etc.), but prior to applying technical measurability thresholds and read-depth normalization rarefactions. The center of each box represents the median value, while the upper and lower limit of the box represent the first and third quartile. The whiskers represent +/- 1.5 times the interquartile range (IQR), and outliers beyond this range are indicated with open circles.

Figure S2

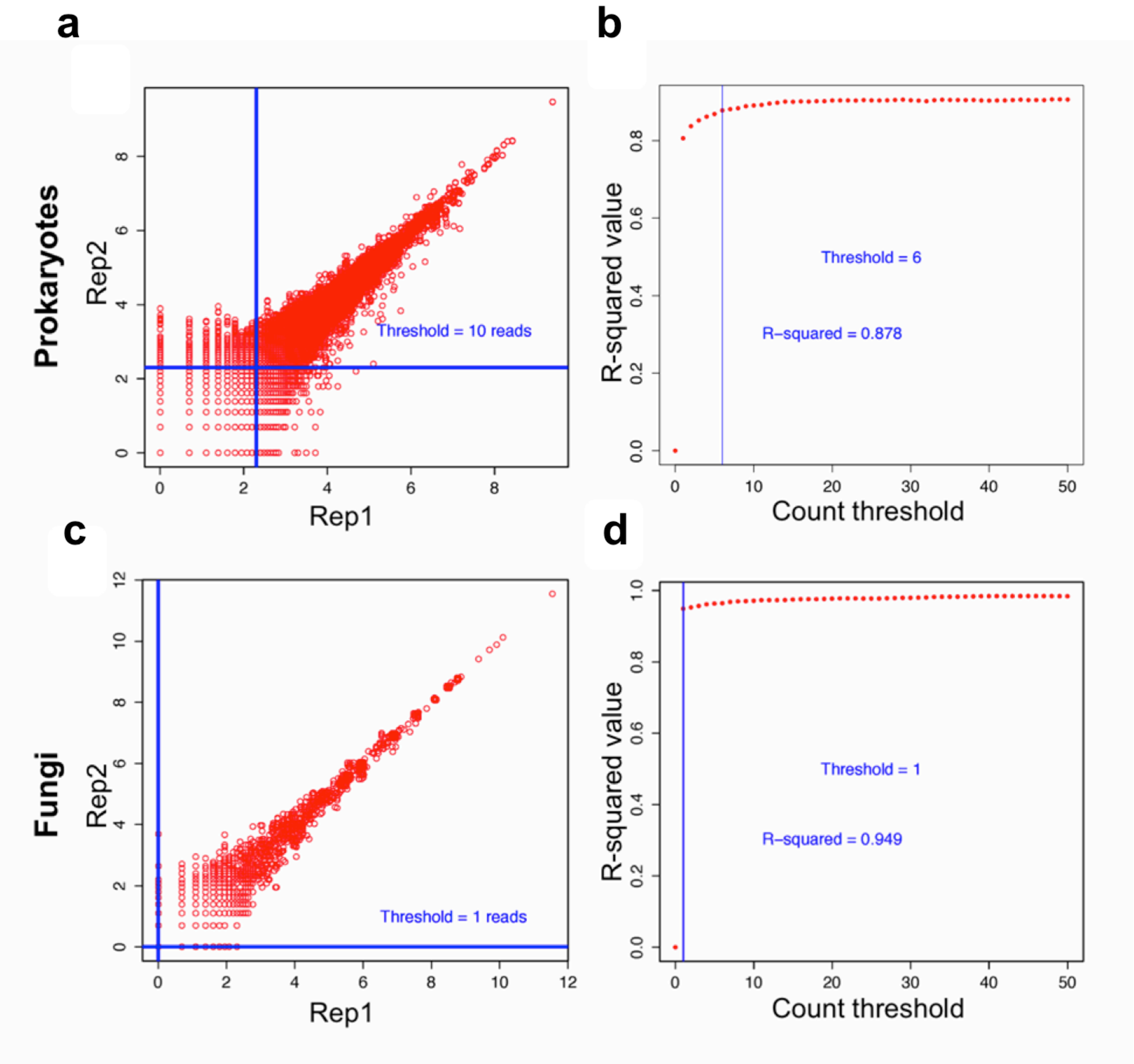

Figure S2. Established cutoff thresholds for technical reproducibility across OTUs for 16S and ITS2 datasets. (**a** and **c**) Plots of log-transformed read counts (in red) for multiple pairs of technical replicates in the prokaryotic (top) and fungal (bottom) datasets. Read counts were first normalized per pair by rarefaction to the number of reads present in the replicate with the lower total read count. (**b** and **d**) Progressive drop-out analysis (as in Lundberg et al. 2013) displaying the R-squared correlation of the data at left as OTUs with low read counts were removed. For the 16S dataset, when only OTUs with > 6 reads are considered (blue line), the R-squared is > 0.90. By contrast, in the ITS2 dataset the R-squared is 0.949 when considering even OTUs with > 1 read count.

Figure S3

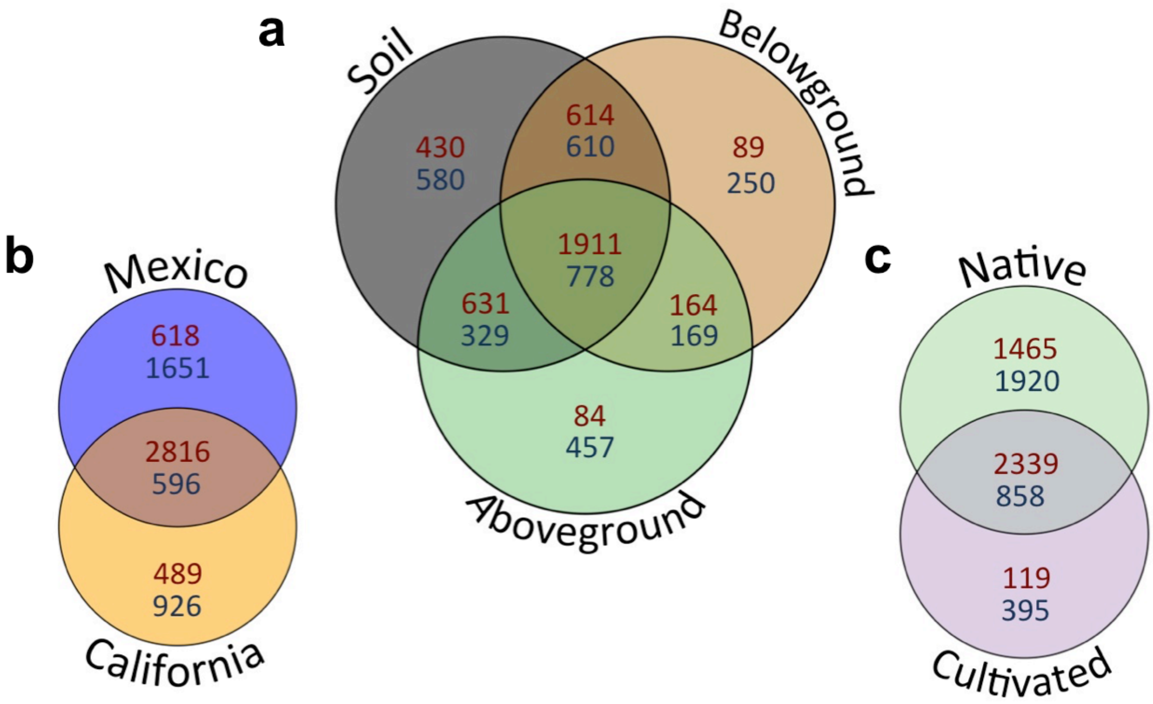

Figure S3. OTU distributions across sample subsets. Venn diagrams indicating the number of prokaryotic (top, in red) and fungal (bottom, in blue) OTUs shared between various subsets of the data, including (**a**) aboveground tissues, belowground tissues, and soil compartments; (**b**) California and Mexico; and (**c**) cultivated and native species.

Figure S4

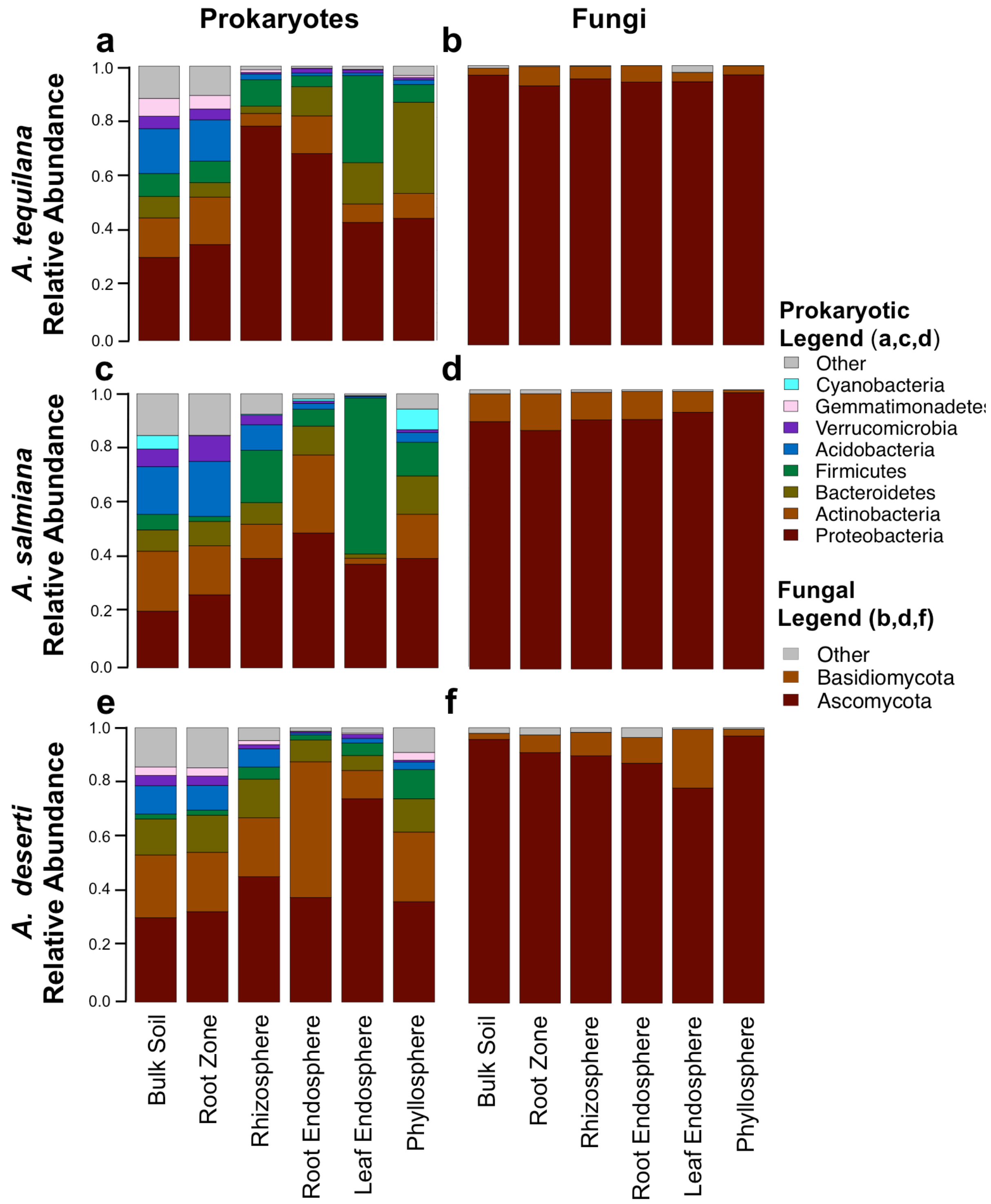

Figure S4. Phylum-level relative abundance plots of prokaryotic and fungal communities associated with each *Agave* species by sample type.

Figure S5

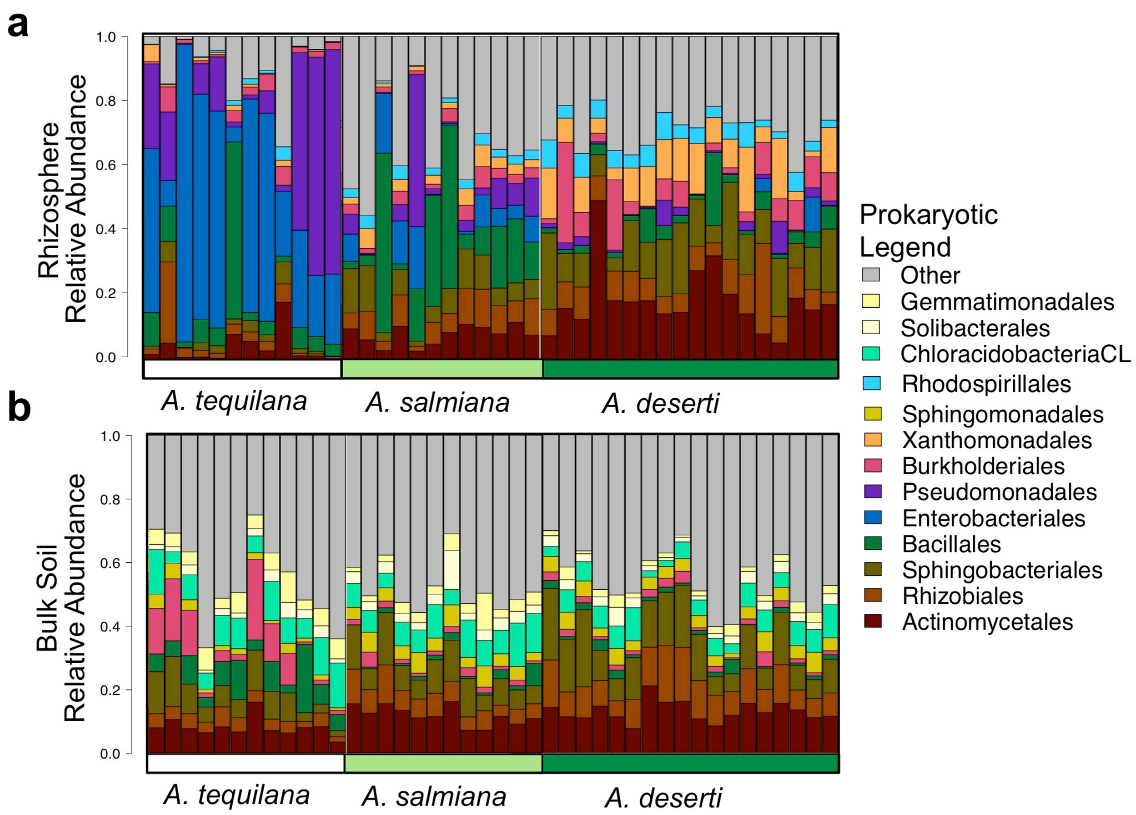

Figure S5. Order-level relative abundance plots of prokaryotic communities associated with all rhizosphere (a) and bulk soil (b) samples, color-coded at bottom by their associated *Agave* species.

Figure S6.

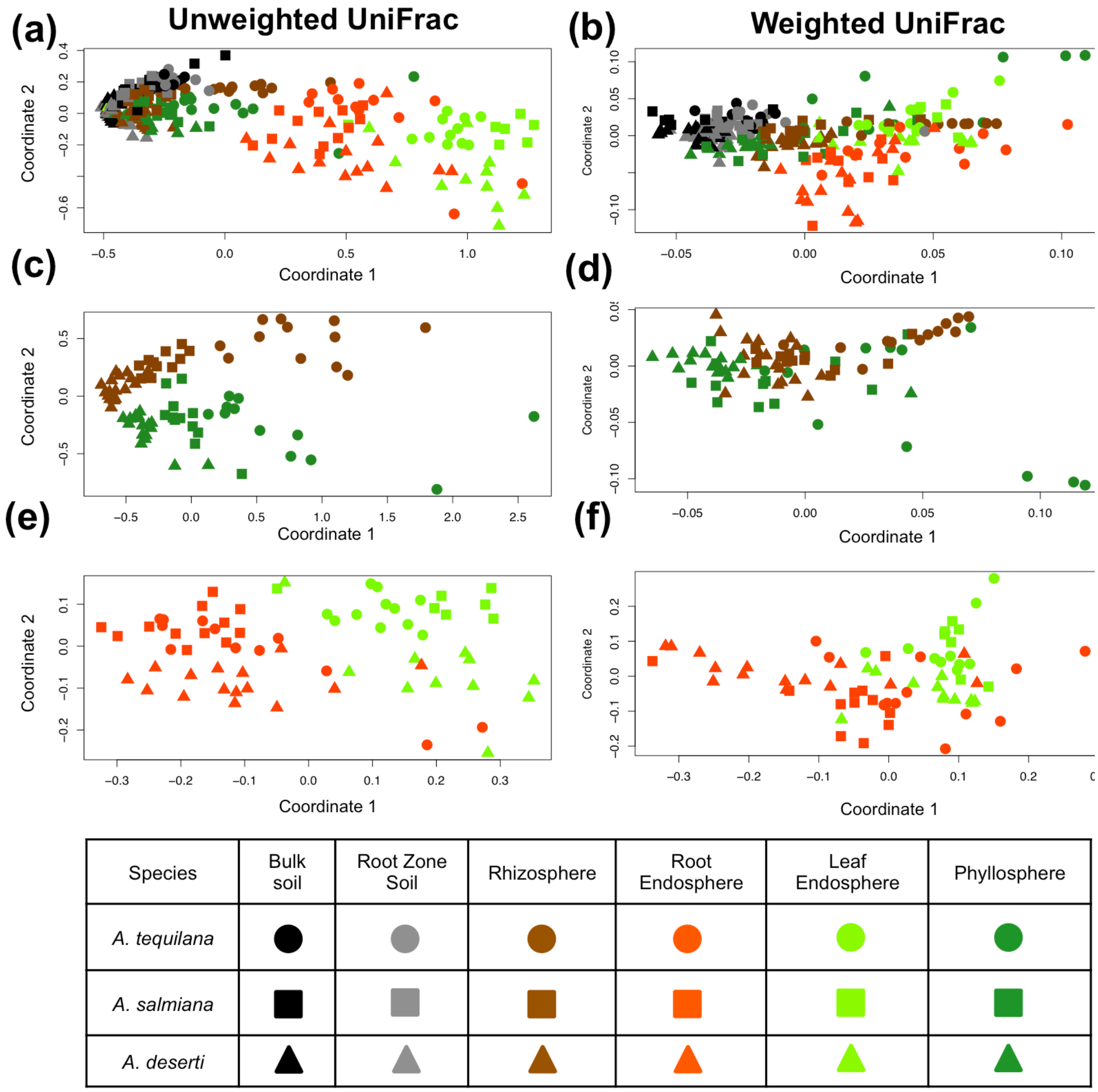

Figure S6. NMDS clustering for prokaryotic data of all samples (a,b), episphere samples (c,d), and endosphere samples (e,f) associated with the three agaves using unweighted (a,c,e) and weighted (b,d,f) UniFrac distances.

Figure S7

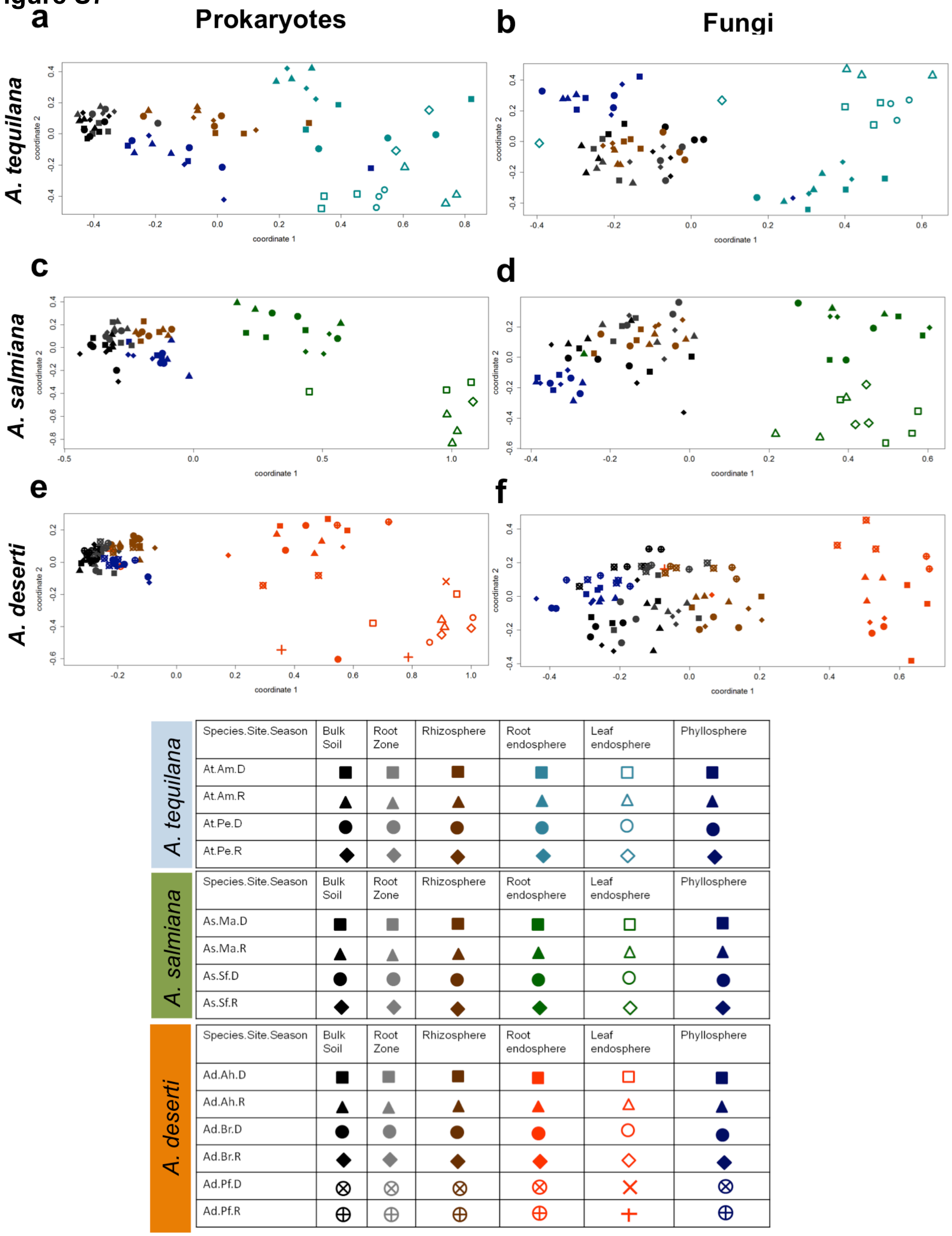

Figure S7. NMDS clustering of all samples (a,b), episphere samples (c,d), and endosphere samples (e,f) associated with the three agaves for prokaryotic (a,c,e) and fungal (b,d,f) communities using Bray Curtis distances. Plots are colored by sample type, host species and location according to the legend.

Figure S8

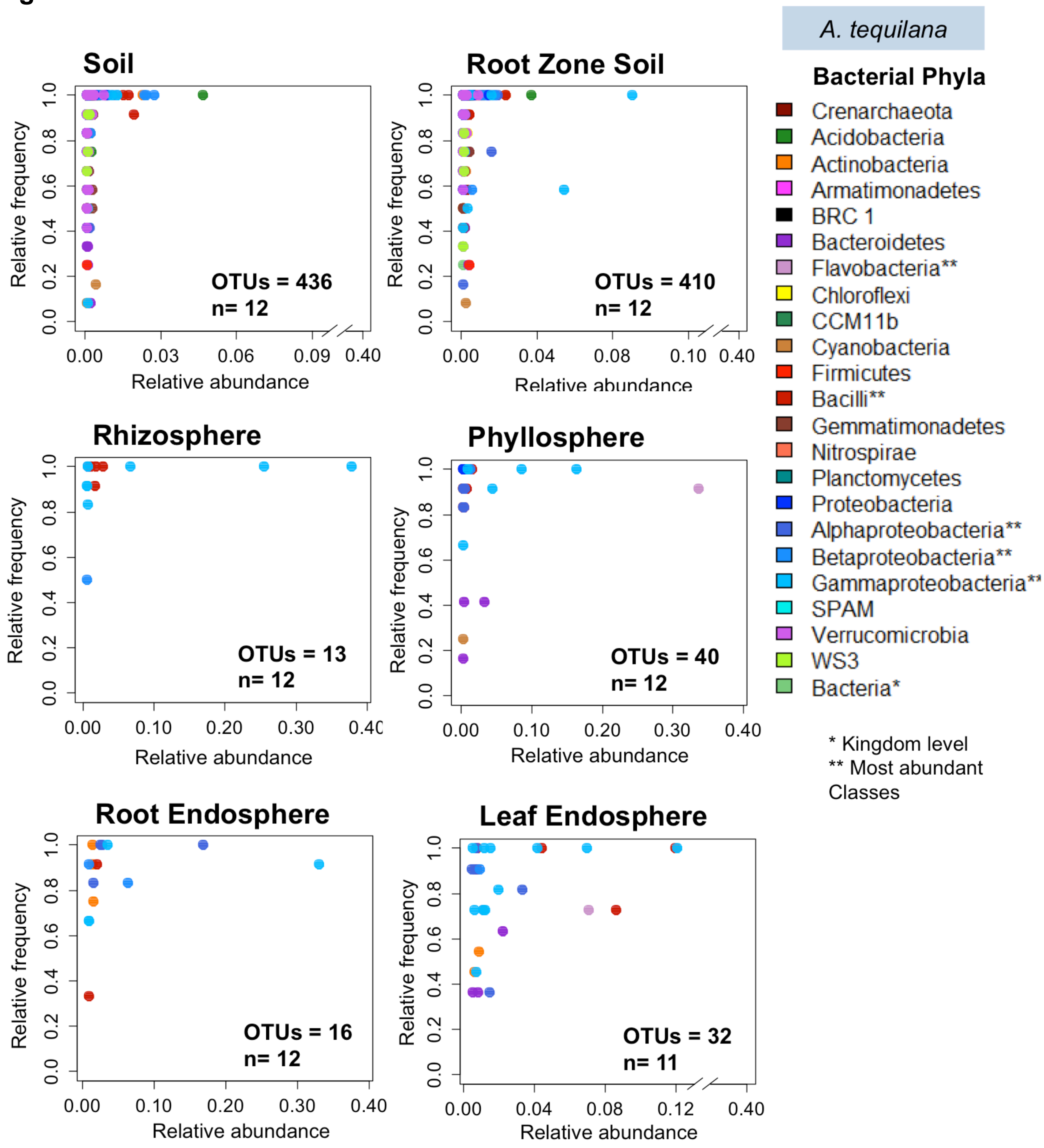

Figure S8. Major prokaryotic taxa associated with *A. tequilana* based on the 80-20 rule (Pareto).

Figure S9

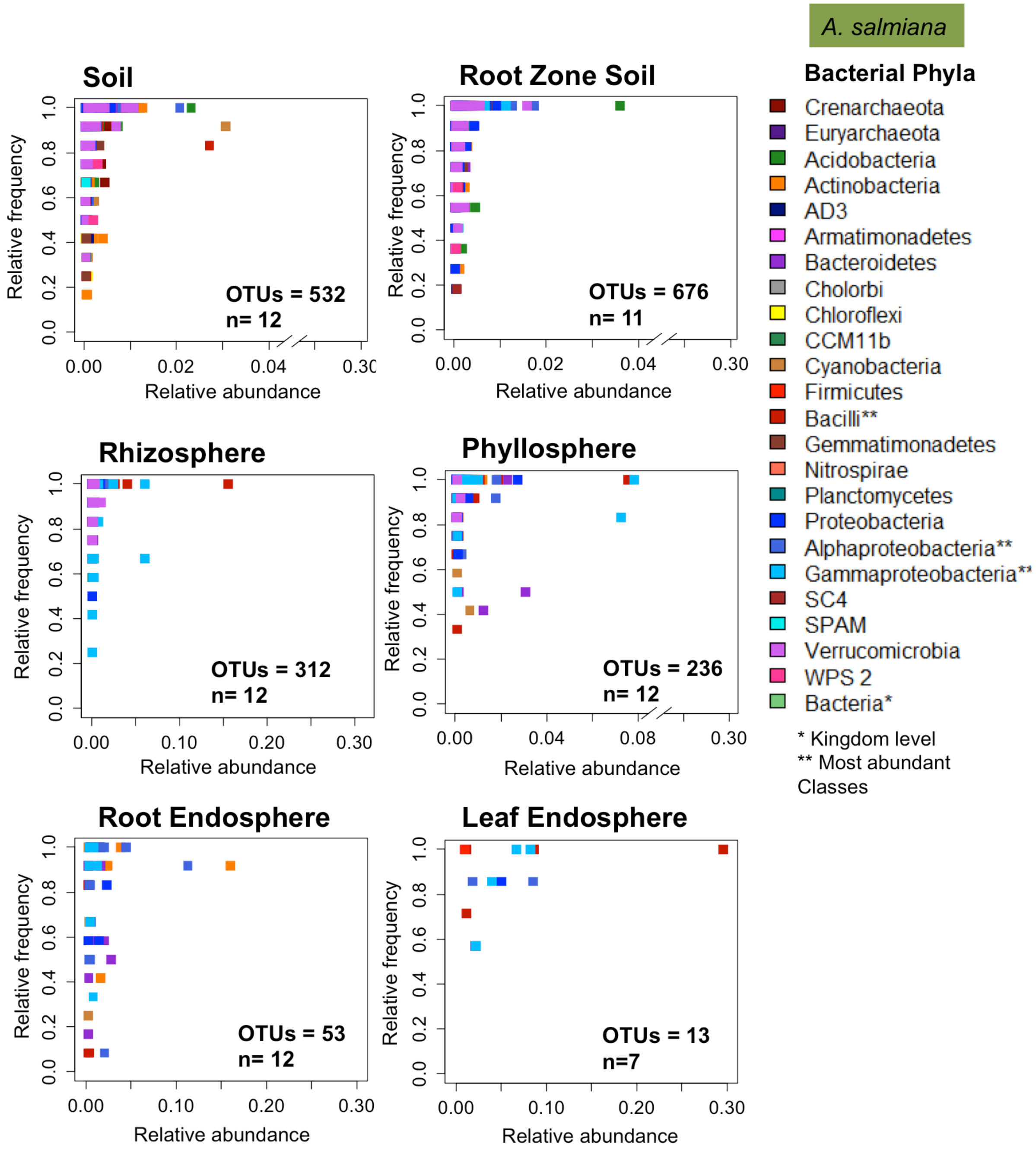

Figure S9. Major prokaryotic taxa associated with *A. salmiana* based on the 80-20 rule (Pareto).

Figure S10.

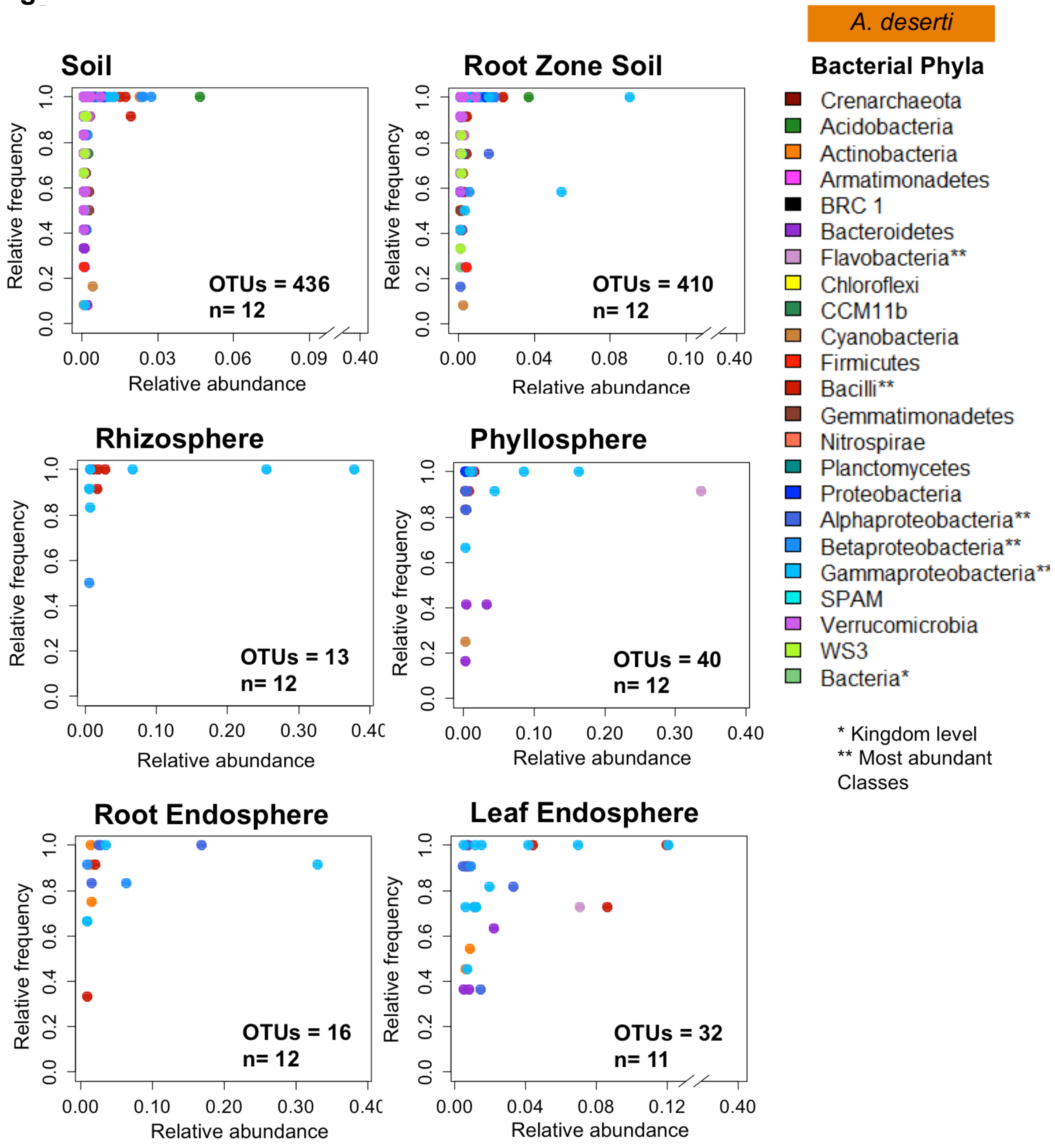

Figure S10. Major prokaryotic taxa associated with *A. deserti* based on the 80-20 rule (Pareto).

Figure S11

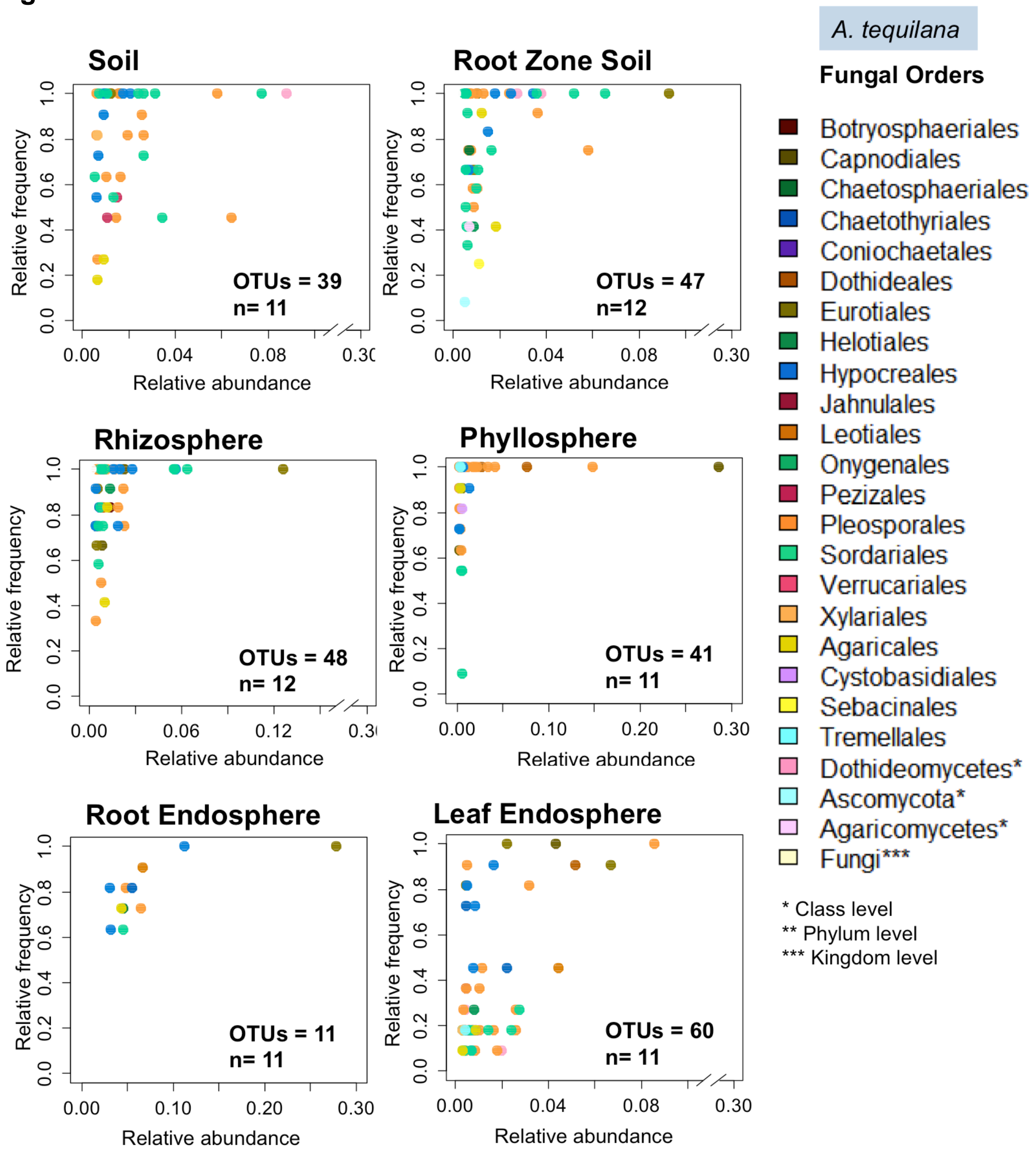

Figure S11. Major fungal taxa associated with *A. tequilana* based on the 80-20 rule (Pareto).

Figure S12.

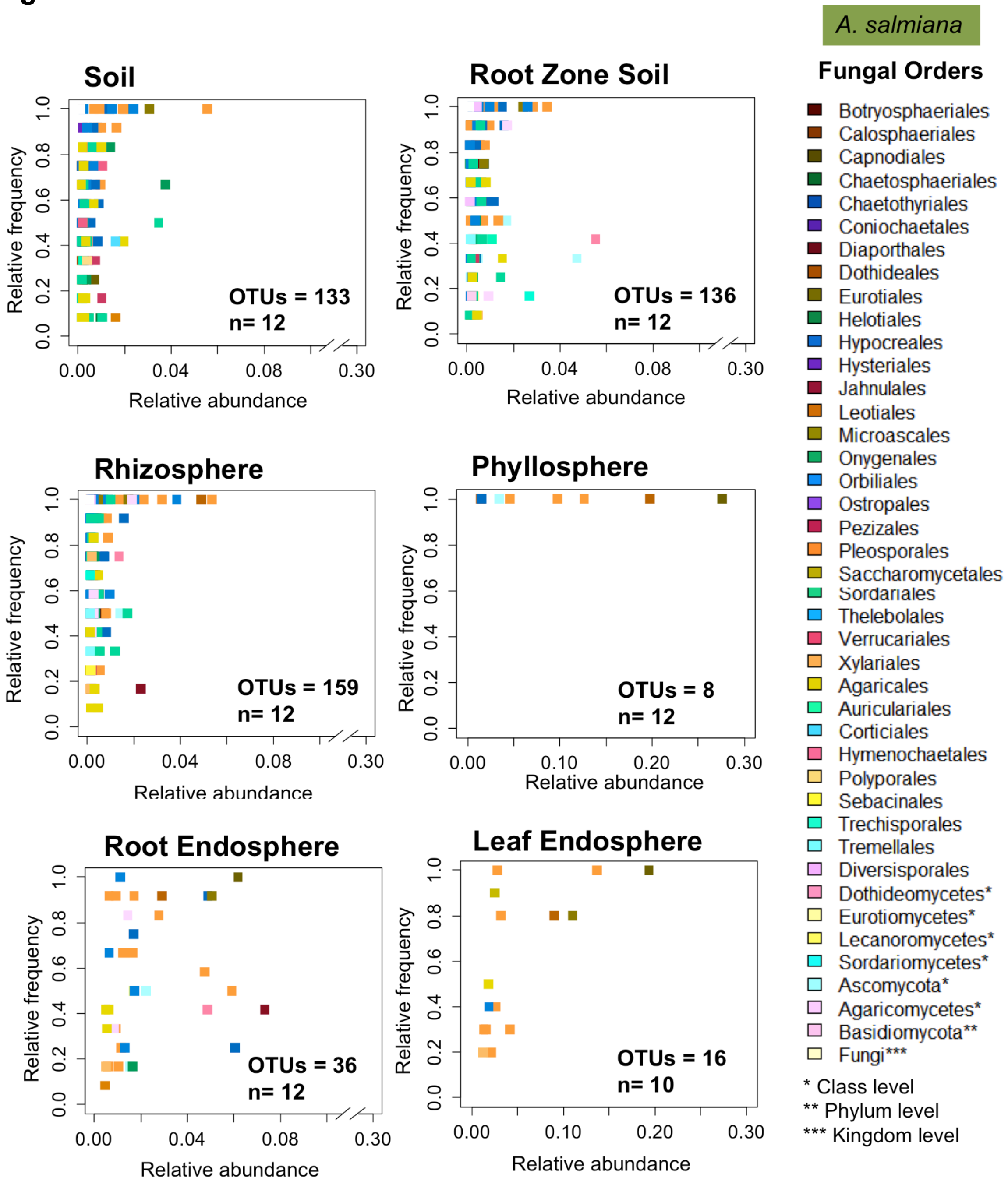

Figure S12. Major fungal taxa associated with *A. salmiana* based on the 80-20 rule (Pareto).

Figure S13

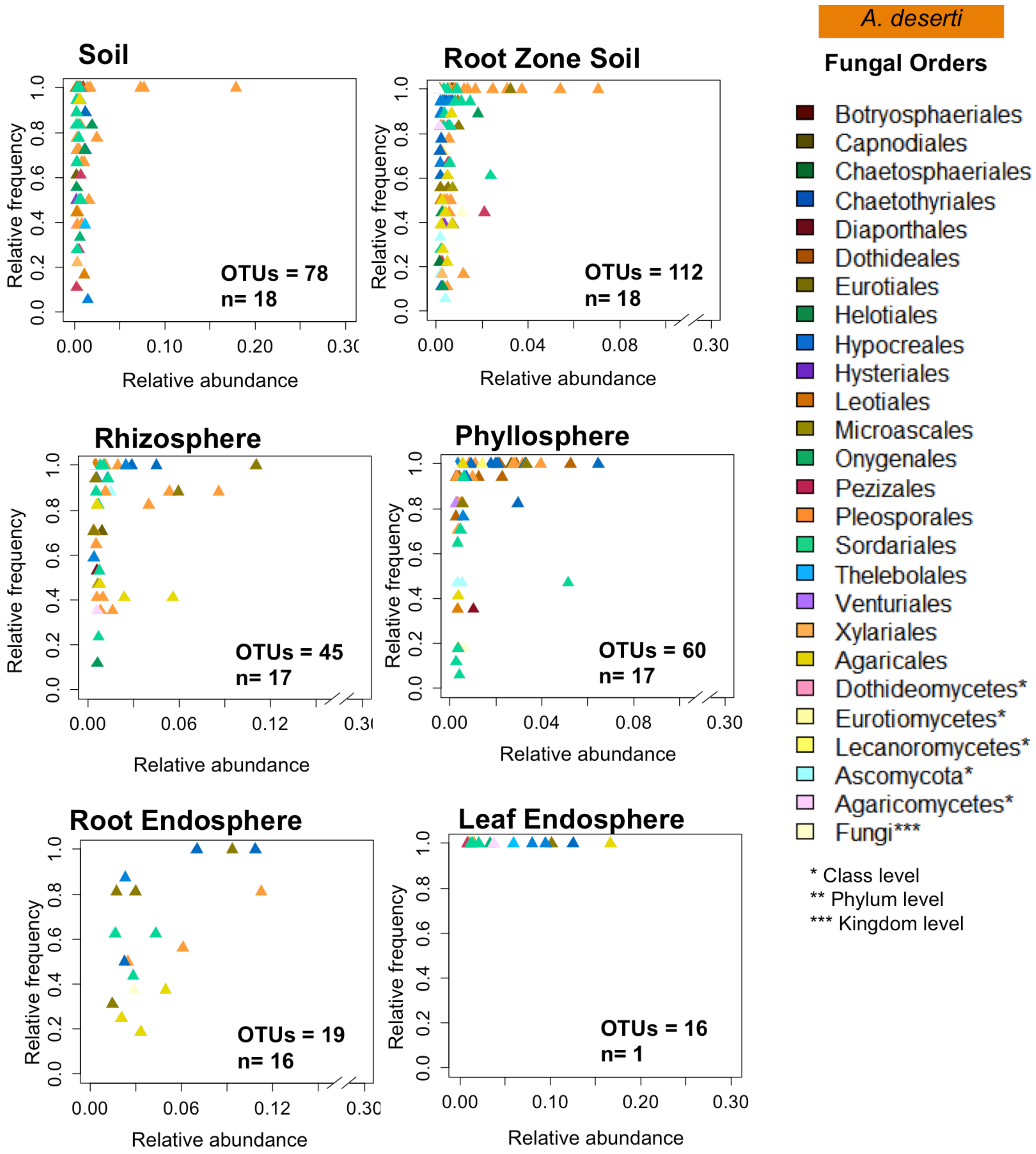

Figure S13. Major fungal taxa associated with *A. deserti* based on the 80-20 rule (Pareto).

Figure S14

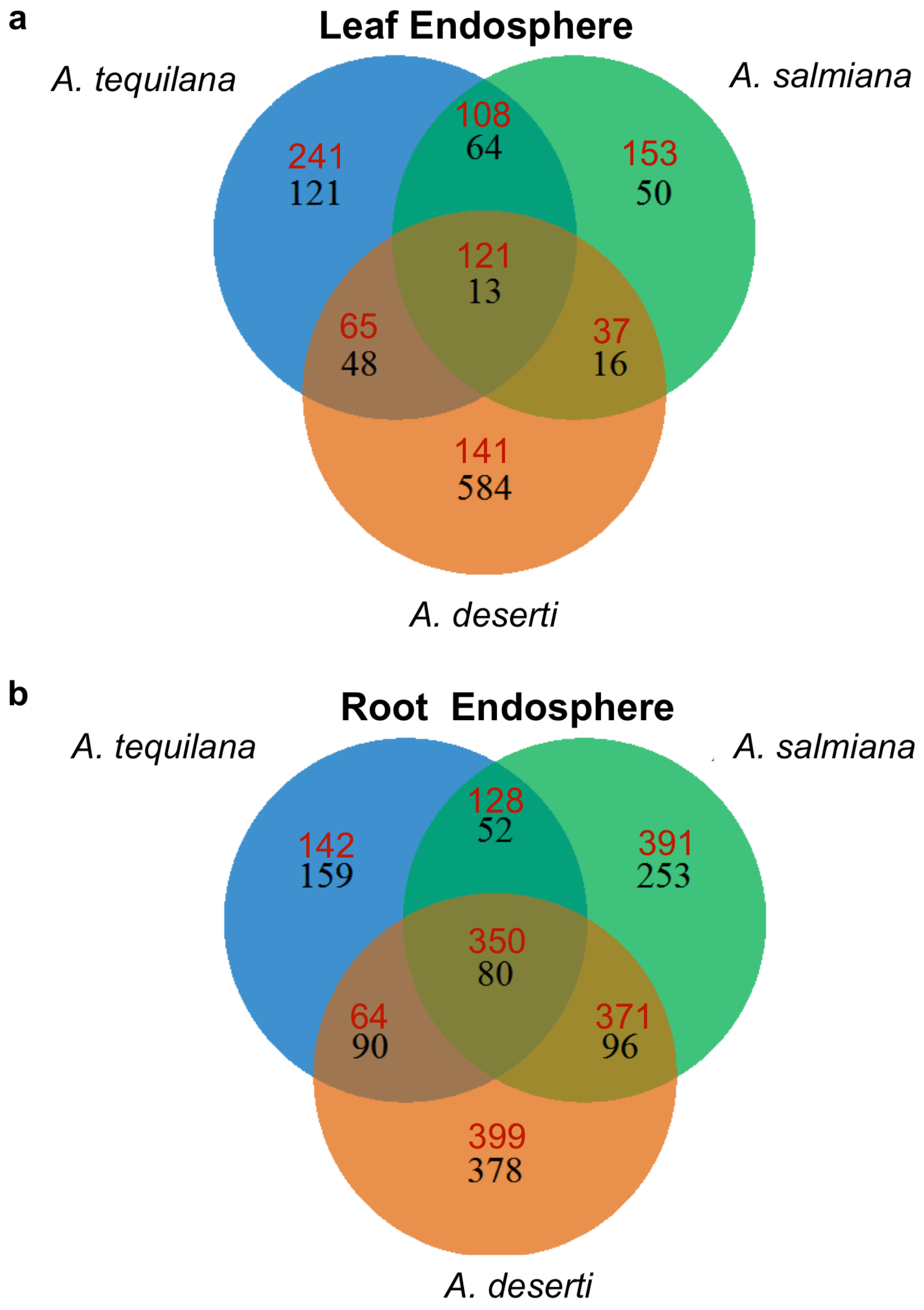

Figure S14. OTU distributions across *Agave* species in the endosphere. Venn diagrams indicating the number of prokaryotic (top, in red) and fungal (bottom, in black) OTUs shared between the three species of Agave in (**A**) the leaf endosphere and (**B**) the root endosphere.

Figure S15

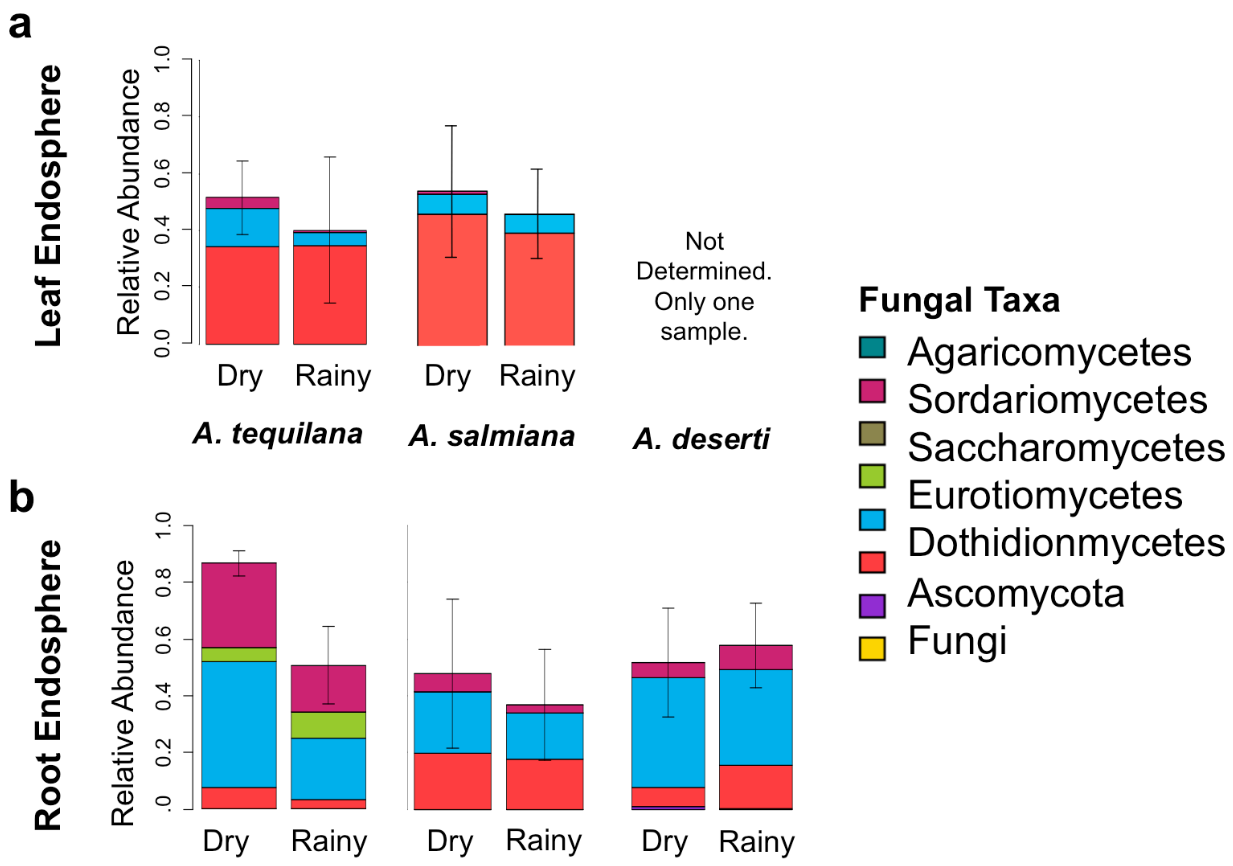

Figure S15. Fungal taxa associated with the endosphere of all three *Agave* species and their relative abundance at the dry and rainy seasons. Relative abundance profiles of the fungal taxa indicated in the legend, +/- the standard deviation (error bars) for the total relative abundance are shown.

Table S1. Mean estimated Shannon diversity with standard deviations in the prokaryotic and fungal communities associated with each *Agave* species.

Sample type	Prokaryotes			Fungi		
	A. tequilana[e]	***A. salmiana***[f]	***A. deserti***[g]	***A. tequilana***[e]	***A. salmiana***[f]	***A. deserti***[g]
**Soil**[a]	5.149 ± 0.324	5.252 ± 0.310	5.303 ± 0.161	2.987 ± 0.431	3.616 ± 0.575	3.109 ± 0.564
**Root Zone Soil**[b]	5.036 ± 0.889	5.516 ± 0.122	5.386 ± 0.242	3.023 ± 0.522	3.574 ± 0.625	3.543 ± 0.618
**Rhizosphere**[c]	2.417 ± 1.211	4.600 ± 1.006	4.803 ± 0.282	3.325 ± 0.391	3.954 ± 0.526	2.751 ± 0.558
**Phyllosphere**[d]	2.716 ± 1.610	4.389 ± 1.039	4.952 ± 0.820	3.052 ± 0.424	2.374 ± 0.528	3.619 ± 0.410
**Root Endosphere**	2.534 ± 1.146	3.316 ± 0.761	2.872 ± 0.644	1.728 ± 0.499	2.139 ± 0.538	1.942 ± 0.448
**Leaf Endosphere**	3.035 ± 0.815	2.051 ± 1.010	2.936 ± 0.763	2.721 ± 0.681	2.225 ± 0.442	3.260

a. Statistically significant difference between species after Kruskal-Wallis test only for Fungi ($H_{2, 41}$ = 6.880, *P*= 0.0321).

b. Statistically significant difference between species after Kruskal-Wallis test for Prokaryotes ($H_{2, 41}$ = 9.458, *P*= 0.0088) and Fungi ($H_{2, 42}$ = 7.507, *P*= 0.0234).

c. Statistically significant difference between species after Kruskal-Wallis test for Prokaryotes ($H_{2, 42}$ = 19.090, *P*= 0.00007) and Fungi ($H_{2, 41}$ = 20.501, *P*= 3.12e^{-05}).

d. Statistically significant difference between species after Kruskal-Wallis test for Prokaryotes ($H_{2, 41}$ = 16.587, *P*= 0.0002) and Fungi ($H_{2, 40}$ = 25.370, *P*= 3.71e^{-06}).

e. Statistically significant difference between sample types associated with *A. tequilana* after Kruskal-Wallis test for Prokaryotes ($H_{5, 71}$ = 42.525, *P*= 4.611e^{-08}) and Fungi ($H_{5, 68}$ = 29.535, *P*= 1.82e^{-05}).

f. Statistically significant difference between sample types associated with *A. salmiana* after Kruskal-Wallis test for Prokaryotes ($H_{5, 66}$ = 42.751, *P*= 4.15e^{-08}) and Fungi ($H_{5, 70}$ = 46.389, *P*= 7.568e^{-09}).

g. Statistically significant difference between sample types associated with *A. deserti* after Kruskal-Wallis test for Prokaryotes ($H_{5, 98}$ = 71.298, *P*= 5.499^{e-14}) and Fungi ($H_{5, 86}$ = 46.242, *P*= 2.193e^{-09}).

Table S2. PERMANOVA of the microbial communities associated with *A. tequilana* considering all factors and their interactions. Only significant factors are displayed ($P \leq 0.05$).

Prokaryotes				Fungi			
**Factor**[a]	**F**	R^2	***P***	**Factor**[a]	**F**	R^2	***P***
Sample Type $_{5,47}$	64.14	0.790	0.001	Sample Type $_{5,44}$	59.901	0.628	0.001
Season $_{1,47}$	4.85	0.012	0.018	Sample Type: Site $_{5,44}$	8.29	0.087	0.001
Site $_{1,47}$	4.76	0.012	0.018	Site $_{1,44}$	37.03	0.078	0.001
				Sample Type: Season: Site $_{5,44}$	4.63	0.049	0.001
				Sample Type: Season $_{5,44}$	3.81	0.040	0.001
				Season $_{1,44}$	6.84	0.014	0.001
				Season: Site $_{1,44}$	5.56	0.012	0.005

a. Numbers in sub-indices indicate the degrees of freedom and residuals of each F test.

Table S3. PERMANOVA of the microbial communities associated with *A. salmiana* considering all factors and their interactions. Only significant factors are displayed ($P \leq 0.05$).

Prokaryotes				Fungi			
**Factor**[a]	**F**	R^2	***P***	**Factor**[a]	**F**	R^2	***P***
Sample Type $_{5,42}$	83.519	0.857	0.001	Sample Type $_{5,46}$	43.675	0.744	0.001
Sample Type: Site $_{5,42}$	2.124	0.022	0.045	Site $_{1,46}$	4.534	0.015	0.004

a. Numbers in sub-indices indicate the degrees of freedom and residuals of each F test.

Table S4. PERMANOVA of the microbial communities associated with *A. deserti* considering all factors and their interactions. Only significant factors are displayed ($P \leq 0.05$).

Prokaryotes				Fungi			
**Factor**[a]	**F**	R^2	***P***	**Factor**[a]	**F**	R^2	***P***
Sample Type $_{5,62}$	78.667	0.810	0.001	Sample Type $_{5,56}$	38.751	0.621	0.001
				Site $_{2,62}$	13.453	0.086	0.001
				Sample Type: Site $_{8,62}$	2.450	0.063	0.002

a. Numbers in sub-indices indicate the degrees of freedom and residuals of each F test.

Table S5. PERMANOVA of the microbial communities associated with the endosphere of *A. tequilana* and *A. salmiana* considering all factors and their interactions. Only significant factors are displayed ($P \leq 0.05$).

Prokaryotes				Fungi			
**Factor**[a]	**F**	R^2	***P***	**Factor**[a]	**F**	R^2	***P***
Sample Type $_{1,34}$	81.186	0.551	0.001	Sample Type $_{1,35}$	40.564	0.390	0.001
Season $_{1,34}$	11.002	0.075	0.001	Species$_{1,35}$	11.572	0.111	0.001
Sample Type: Season $_{1,34}$	6.287	0.043	0.004	Sample Type: Species$_{1,35}$	6.039	0.058	0.004
Season: Species$_{1,34}$	5.662	0.038	0.008	Season$_{1,35}$	4.503	0.043	0.008
Sample Type: Species$_{1,34}$	3.428	0.023	0.037	Sample Type: Season: Species $_{1,35}$	2.756	0.026	0.044
Species$_{1,34}$	3.497	0.024	0.038	Sample Type: Season$_{1,35}$	2.240	0.022	0.050

a. Numbers in sub-indices indicate the degrees of freedom and residuals of each F test.
